# Functional activity of the H3.3 histone chaperone complex HIRA requires trimerization of the HIRA subunit

**DOI:** 10.1038/s41467-018-05581-y

**Published:** 2018-08-06

**Authors:** Dominique Ray-Gallet, M. Daniel Ricketts, Yukari Sato, Kushol Gupta, Ekaterina Boyarchuk, Toshiya Senda, Ronen Marmorstein, Geneviève Almouzni

**Affiliations:** 1 Institut Curie, PSL Research University, CNRS, UMR3664, Equipe Labellisée Ligue contre le Cancer, F-75005 Paris, France; 2 Sorbonne Université, Institut Curie, CNRS, UMR3664, F-75005 Paris, France; 3 Department of Biochemistry and Biophysics, Graduate Group in Biochemistry and Molecular Biophysics, Abramson Family Cancer Research Institute, Perelman School of Medicine at the University of Pennsylvania, Philadelphia, PA 19104 USA; 4 Structural Biology Research Center (SBRC), Institute of Materials Structure Science (IMSS), High Energy Accelerator Research Organization (KEK), 1-1 Oho, Tsukuba, Ibaraki 305-0801 Japan; 5 Department of Biochemistry and Biophysics, Perelman School of Medicine at the University of Pennsylvania, Philadelphia, PA 19104 USA; 6 Department of Materials Structure Science, School of High Energy Accelerator Science, The Graduate University for Advanced Studies (Soken-dai), 1–1 Oho, Tsukuba, Ibaraki 305–0801 Japan; 7 Present Address: Graduate School of Life Sciences, Tohoku University, 2-1-1, Katahira Aoba-ku, Sendai, Miyagi 980-8577 Japan

## Abstract

The HIRA histone chaperone complex deposits the histone variant H3.3 onto chromatin in a DNA synthesis-independent manner. It comprises three identified subunits, HIRA, UBN1 and CABIN1, however the functional oligomerization state of the complex has not been investigated. Here we use biochemical and crystallographic analysis to show that the HIRA subunit forms a stable homotrimer that binds two subunits of CABIN1 in vitro. A HIRA mutant that is defective in homotrimer formation interacts less efficiently with CABIN1, is not enriched at DNA damage sites upon UV irradiation and cannot rescue new H3.3 deposition in HIRA knockout cells. The structural homology with the homotrimeric replisome component Ctf4/AND-1 enables the drawing of parallels and discussion of the functional importance of the homotrimerization state of the HIRA subunit.

## Abstract (web summary)

The HIRA histone chaperone complex is involved in the deposition of the histone variant H3.3. Here the authors, by using biochemical and crystallographic approaches, report the homotrimerization of the HIRA subunit which is critical for the functional activity of the complex.

## Introduction

Histone chaperones are currently considered the most likely candidates responsible for histone variant deposition^1,2^. The presence of the Histone regulator A (HIRA) with the histone variant H3.3 in a soluble complex has implicated this histone chaperone in the specific deposition of this variant^3^. HIRA promotes histone deposition independently of DNA synthesis as shown in *Xenopus laevis* egg extracts^4^ and deposits new histones H3.3 in both replicating and non-replicating human cells^5^. HIRA is in a complex that contains at least two other subunits, Calcineurin-binding protein 1 (CABIN1) and Ubinuclein 1 (UBN1)^6^. Notably, these three components have counterparts in yeast that constitute the HIR complex^7^. In addition to the HIRA complex, other histone chaperones, in particular Anti-silencing factor 1 (ASF1a and ASF1b), have been purified with H3.3^3^. ASF1a interacts specifically with the HIRA subunit connecting this chaperone with the DNA synthesis-independent assembly pathway mediated by the HIRA complex^8,9^. The HIRA-mediated H3.3 deposition has been strongly associated with actively transcribed genes^10^. Moreover, it has been involved in either the activation or the long-term maintenance of gene expression patterns^11,12^ and HIRA depletion has been reported to affect transcription recovery after DNA repair^13^.

Within the HIRA complex, a Hpc2-related domain (HRD) of UBN1 interacts directly with H3.3 as revealed by in vitro biochemical studies and X-ray crystallography^14^. Notably, in addition to UBN1, another Ubinuclein exists, UBN2, that is also able to interact with HIRA^15^. However, whether UBN1 and UBN2 are present together in the same complex or constitute distinct entities is not known. CABIN1, originally identified as a Calcineurin-binding protein whose interaction is dependent on protein kinase C (pKC) and calcium signals, negatively regulates the activity of other interactors, such as Myocyte enhancer factor 2 (MEF2) and p53^16–18^. However, its specific function in the HIRA complex remains to be determined. Furthermore, HIRA serves as a scaffold protein to bring together the other subunits as well as ASF1a. It binds UBN1/2, CABIN1, and ASF1a through its N-terminal WD40 repeat, C-terminal C, and central B domains, respectively^8,15,19^. Depletion of HIRA leads to the co-depletion of both CABIN1 and UBN1 proteins further underlining the fact that HIRA plays a central role^5^. The DNA-binding capacity of the complex has been proposed to promote a general recruitment at any transient nucleosome-free region for H3.3 deposition^5,20^. The fact that the HIRA complex associates with various proteins, such as transcription factors, RNA pol II, Prohibitin (PHB), and replication protein A (RPA) suggests that the targeting of the complex at specific locations may exploit additional structural properties and mechanisms^5,21–24^. Finally, the H3.3 deposition activity of the complex has been reported to be modulated by post-translational modifications of the HIRA subunit^25,26^.

Considering the central position of HIRA, and its multiple partners, a major question to address remained despite these studies, namely to decipher the functional oligomerization state of the HIRA complex. By using biochemical and structural approaches, our study reveals that the HIRA subunit forms a homotrimer with homology to the replisome component Cohesion establishment factor 4/Acidic nucleoplasmic DNA-binding protein-1 (Ctf4/AND-1). This homotrimerization is critical for CABIN1 interaction, which binds with a 3:2 HIRA–CABIN1 stoichiometry in vitro and key for HIRA enrichment at UV damage sites and for efficient new H3.3 deposition. We thus provide a quaternary structure of the HIRA subunit with major implications for the functional activity of the complex.

## Results

### The HIRA subunit homooligomerizes in cells

**Fig. 1:**
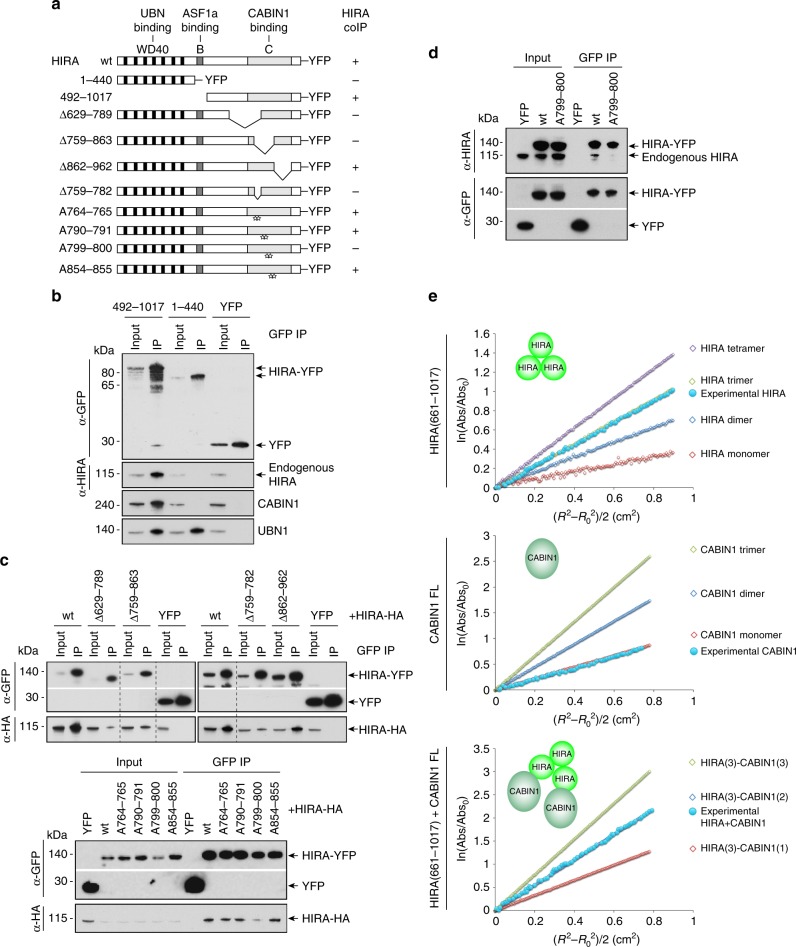
HIRA homooligomerizes in cells and forms a homotrimer in vitro. **a** YFP constructs of human HIRA and mutants. The WD40 repeat (aa 1–369), B (aa 439–475) and C (aa 763–963) domains involved in UBN, ASF1a, and CABIN1 interactions, respectively, are shown. Star indicates single amino acid substitution with alanine. The co-immunoprecipitation efficiency of endogenous HIRA or HIRA-HA (HIRA coIP) is indicated for each construct, “+” indicates that the efficiency of coIP is similar to the one obtained with the wt HIRA construct and “−” indicates that the efficiency of the coIP is decreased. **b** Western blot analysis of anti-GFP-immunoprecipitates from U2OS nuclear extracts expressing YFP-tagged proteins. **c** Western blot analysis of anti-GFP-immunoprecipitates from U2OS nuclear extracts expressing both YFP-tagged and HIRA-HA proteins. **d** Western blot analysis of anti-GFP-immunoprecipitates from U2OS nuclear extracts expressing YFP-tagged proteins. In **b**, **c** and **d**, input corresponds to 10% of nuclear extract used for each experiment. **e** Equilibrium sedimentation of recombinant proteins HIRA(661–1017), CABIN1 full length (FL) and HIRA(661–1017) + CABIN1 FL. Theoretical (open symbols) and experimental (closed symbol) curves are shown

In order to explore the interaction of human HIRA with its known co-subunits, we generated two YFP-tagged HIRA constructs corresponding to the amino acids 1–440 and 492–1017 that contain the interaction domains for UBN1 and CABIN1, respectively^15,19^ (Fig. 1a). We performed immunoprecipitation with extracts from U2OS-transfected cells using anti-GFP beads (that recognize YFP-tagged proteins) and found as expected that UBN1 and CABIN1 co-immunoprecipitated, with HIRA(1–440) and HIRA(492–1017), respectively (Fig. 1b). However, unexpectedly, UBN1 also co-immunoprecipitated with a HIRA C-terminal construct in which the UBN1 interaction region was missing, HIRA(492–1017). Moreover, we found that endogenous HIRA co-immunoprecipitated with HIRA(492–1017) but not with HIRA(1–440) suggesting a possible homooligomerization of HIRA through its C-terminal half. To test this hypothesis and to localize the domain of HIRA involved in its self-interaction, we generated additional constructs expressing a series of deletion mutants of the HIRA protein (Fig. 1a), we co-expressed each of them with a HIRA-HA construct in U2OS cells and performed anti-GFP immunoprecipitation with the derived cell extracts. While the deletion Δ862–962 did not affect HIRA-HA co-immunoprecitation as compared to the wt protein, we observed that the deletions Δ629–789, Δ759–863, and Δ759–782 decreased the amount of co-immunoprecipitated HIRA-HA (Fig. 1c). This confirmed that a domain in the C-terminal half part of HIRA could be involved in a homooligomerization. To identify potential amino acids critical for HIRA homooligomerization, we examined a sequence alignment of the HIRA protein from different species to identify conserved amino acids (Supplementary Fig. 1b). We generated four double amino acid mutants, changing highly conserved amino acids with alanines (Fig. 1a). Among these four mutants, HIRA(W799A–D800A) exhibited a reduction in the capacity to co-immunoprecipitate HIRA-HA, supporting a role for amino acids W799 and/or D800 in the homooligomerization of HIRA (Fig. 1c). Furthermore, this HIRA(W799A–D800A) mutant failed to co-immunoprecipitate endogenous HIRA as compared to HIRA wt (Fig. 1d). Our results revealed that the HIRA subunit homooligomerizes in cells through a region in the C-terminal half of the protein and we also delineated the critical amino acids Trp799 and/or Asp800 in this region.

### The HIRA subunit forms a homotrimer in vitro

We analyzed the capacity of HIRA to homooligomerize in vitro using a recombinant human HIRA protein (aa 661–1017) produced in bacteria. This HIRA construct was selected based on secondary structure prediction and sequence conservation among HIRA homologs (Supplementary Fig. 1a, b; Supplementary Methods). We demonstrated that HIRA(661–1017) forms a stable monodisperse population eluting between the 158 and 44 kDa standards on size-exclusion chromatography, consistent with homooligomer formation (Supplementary Fig. 3). The size-exclusion peak was subjected to analytical ultracentrifugation to determine the molecular assembly. Sedimentation equilibrium analysis was performed using purified HIRA(661–1017) (Supplementary Fig. 2a), and single species fits using the programs Heteroanalysis and SEDPHAT indicated solution molecular weights of ~117 and ~115 kDa, respectively, most consistent with a homotrimer of theoretical molecular weight of 118,710 Da (Fig. 1e; Supplementary Fig. 2b, c; Supplementary Table 1). Further analysis with SEDPHAT using a monomer–trimer equilibrium model provides the best description of the data, with a determined *K*_d_^1–3^ of 3.6 μM although *K*_d_^1–3^ values ranging from 0.3 to 10 μM also provided acceptable quality of fits (Supplementary Fig. 2c; Supplementary Table 2), leading us to the conclusion that the HIRA *K*_d_^1–3^ lies within the range of 300 nM to 10 μM. Sedimentation velocity experiments also supported a single species trimer (*S*_T,b_ = 5.6 and *f/f*_o_ = 1.47), consistent with the calculated properties of the HIRA(680–1017) crystal structure (5.8*S*, *f/f*_o_ of 1.24) (Supplementary Fig. 2d).

Having shown that HIRA is able to homooligomerize in vitro as in cells, we examined if Δ759–782 deletion and W799A–D800A double amino acid mutants also exhibit a reduction in the formation of homooligomer in vitro. However, biochemical analysis showed that both mutants eluted entirely in the void volume of a size-exclusion column (Supplementary Fig. 3) consistent with protein misfolding and aggregation. Based on this data, we conclude that the HIRA subunit homooligomerizes and forms a stable homotrimer in vitro.

### The HIRA homotrimer binds two CABIN1 subunits in vitro

The C-terminal half of the HIRA protein containing the C domain was previously shown to be involved in CABIN1 interaction^19^. We aimed to determine the stoichiometry of CABIN1 alone and when associated with HIRA. Sedimentation equilibrium analysis was carried out for the full-length (FL) CABIN1 protein produced in Sf9 cells. From single species fitting using both Heteroanalysis and SEDPHAT we observed that CABIN1 formed a monomer in solution with experimental and theoretical molecular weights of ~245 kDa and 246,352 Da, respectively (Fig. 1e; Supplementary Fig. 2a-c; Supplementary Table 1). Sedimentation velocity analysis of CABIN1 FL revealed a single species with a *S*_T,b_ = 6.6 and *f/f*_o_ = 2.0, consistent with an highly elongated monomer in solution (Supplementary Fig. 2d).

The HIRA–CABIN1 complex isolated from a size-exclusion peak of the complex was analyzed in similar sedimentation equilibrium experimental conditions but with slightly lower rotor speeds. Formally, a trimer of HIRA would have upwards of three possible binding sites for CABIN1. However, the experimental molecular weight determined from single species fitting of the data was substantially less than that for a predicted 3:3 complex, with experimental molecular weights of ~550 and ~606 kDa determined from SEDPHAT and Heteroanalysis, respectivley (Fig. 1e; Supplementary Fig. 2b,c). These molecular weights were most consistent with a HIRA(3)–CABIN1(2) complex with a theoretical molecular weight of 610,982 Da. Further analysis with SEDPHAT showed that the data is well-described by an ABBB global fitting model with three symmetric sites and a macroscopic *K*_d_, where A is the HIRA trimer and B is a monomer of CABIN1 (Supplementary Fig. 2c; Supplementary Table 1). While the apparent *K*_d_ values for the first two binding events have apparent tight binding affinities < 1 nM, far below the experimental concentrations of sample, the third binding event is modeled with an appreciably weaker association constant of ~10 μM (which is well above the submicromolar concentrations of sample in this experiment) (Supplementary Fig. 2c; Supplementary Table 1). The data could be comparably well fit to a ABB association model with comparable tight binding, supporting formation of a HIRA(3)–CABIN1(2) complex (Fig. 1e; Supplementary Fig. 2b). Sedimentation velocity analysis of the HIRA–CABIN1 complex revealed a single 12.2 *S*_T,b_ species with a *f/f*_o_ of 1.99, consistent with a HIRA(3)–CABIN1(2) stoichiometry (Supplementary Fig. 2d). We also performed sedimentation velocity analysis on HIRA(661–1017), CABIN1 FL and the HIRA–CABIN1 complex in buffer containing 150 mM NaCl in an effort to observe the oligomerization state of the proteins under more physiologically relevant conditions (Supplementary Fig. 2d). All *S*_T,b_ and *f/f*_o_ values observed at 150 mM NaCl are consistent with values determined under experimental conditions with 300 mM NaCl. This data suggests that the homotrimeric HIRA binds two subunits of CABIN1 in vitro.

### HIRA homotrimerization is critical for CABIN1 binding

As HIRA has been previously described as a long-lived protein^27^, we explored whether the homooligomerization of HIRA may play a role in its protein stability. However, we did not observe any difference between the wt and (W799A–D800A) homotrimerization mutant proteins upon cycloheximide treatment (Supplementary Fig. 4b), arguing that the homooligomerization of HIRA is not involved in the stability of the protein.

**Fig. 2:**
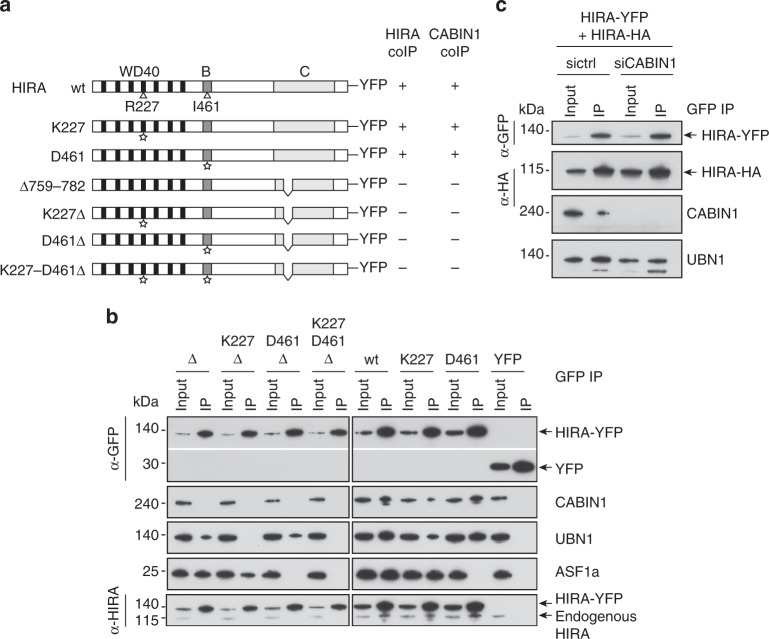
HIRA homooligomerization is required for CABIN1 interaction. **a** YFP constructs of human HIRA and mutants. The amino acids R227 and I461 critical for UBN and ASF1a interactions, respectively, are shown. Star indicates single amino acid substitution (R227 with K or I461 with D). The co-immunoprecipitation efficiencies of both endogenous HIRA (HIRA coIP) and CABIN1 (CABIN1 coIP) are indicated for each construct, “+” indicates that the efficiency of coIP is similar to the one obtained with the wt HIRA construct and “−” indicates that the efficiency of the coIP is decreased. **b** Western blot analysis of anti-GFP-immunoprecipitates from U2OS nuclear extracts expressing YFP-tagged proteins. **c** Western blot analysis of anti-GFP-immunoprecipitates from U2OS nuclear extracts expressing both HIRA-YFP and HIRA-HA proteins prepared from cells treated with siRNAs control or CABIN1. In **b** and **c**, input corresponds to 10% of nuclear extract used for each experiment

To study the impact of HIRA homooligomerization on the interaction with key partners in cells, we generated several HIRA-YFP constructs with single amino acid substitution known to affect the binding of UBN, HIRA(R227K)^15^, or ASF1a, HIRA(I461D)^8^, and also combined them with the deletion mutant HIRA(Δ759–782) that affects HIRA homooligomerization (Fig. 2a). We used extracts from transfected U2OS cells for anti-GFP immunoprecipitation. As expected, the ASF1a protein was undetectable in the immunoprecipitation with HIRA(I461D) (Fig. 2b). However, UBN1 remained co-immunoprecipitated with HIRA(R227K), yet in a reduced amount when compared with the wt protein and not detected at all with HIRA(Δ759–782 + R227K) (Fig. 2b). Interestingly, we found that CABIN1, but not ASF1a or UBN1, was lost in the immunoprecipitation with HIRA(Δ759–782) as compared to the wt protein (Fig. 2b). These data argue for a scheme in which a HIRA protein that does not homooligomerize cannot properly interact with CABIN1. We further confirmed this finding with the amino acids homooligomerization mutant HIRA(W799A/D800A) (Supplementary Fig. 4a). Although we showed that HIRA alone can homooligomerize in vitro (Fig. 1e), we wanted to know whether the presence of CABIN1 could be required for HIRA homooligomerization in cells. To test this, we depleted CABIN1 by siRNA interference and we co-transfected HIRA-YFP and HIRA-HA. By anti-GFP immunoprecipitation, we observed co-immunoprecipitation of HIRA-HA with HIRA-YFP in similar amount when using extracts derived from U2OS cells treated either with the siCont or siCABIN1, indicating that affecting CABIN1 dosage does not impact HIRA self-interaction in cells (Fig. 2c). Our results show that preventing HIRA homooligomerization has a major impact on its capacity to interact with CABIN1.

### CABIN1 binding and homotrimerization domains are distinct

**Fig. 3:**
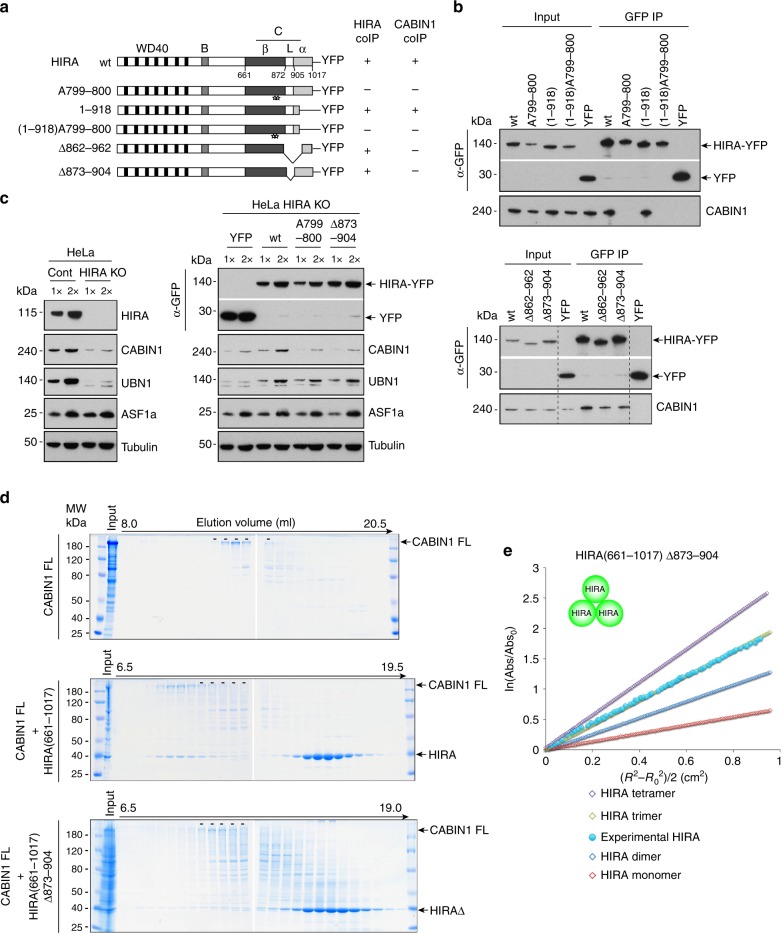
Domains of HIRA involved in its homooligomerization and CABIN1 interaction are distinct. **a** YFP constructs of human HIRA and mutants. Three different structures matching the previously described C domain are indicated: β-strand (aa 661–872), loop (aa 873–904) and α-helical (aa 905–1017) domains. Star indicates single amino acid substitution with alanine. The co-immunoprecipitation efficiency of endogenous CABIN1 (CABIN1 coIP) is indicated for each construct, “+” indicates that the efficiency of coIP is similar to the one obtained with the wt HIRA construct and “−” indicates that the efficiency of the coIP is decreased. **b** Western blot analysis of anti-GFP-immunoprecipitates from U2OS nuclear extracts expressing YFP-tagged proteins. Input corresponds to 10% of nuclear extract used for each experiment. **c** (Left) Western blot analysis of nuclear extracts from HIRA KO and control HeLa cells. (Right) Western blot analysis of nuclear extracts from HeLa HIRA KO cells expressing YFP-tagged proteins. **d** Superose 6 fractionation of recombinant proteins CABIN1 FL, CABIN1 FL + HIRA(661–1017) and CABIN1 FL + HIRA(661–1017) Δ873–904. The dashes indicate the fractions in which free CABIN1 FL elutes. Input corresponds to protein at the concentration that it was loaded onto the Superose 6 column. **e** Equilibrium sedimentation of recombinant protein HIRA(661–1017) Δ873–904. Theoretical (open symbols) and experimental (closed symbol) curves are shown

CABIN1 has been previously reported to interact with the HIRA C domain^19^ that matches the domain that we identified as key for HIRA homooligomerization. However, in contrast to ASF1a and UBN1, a defined interaction involving critical amino acids has not been determined for CABIN1. We therefore wondered whether the precise domain of HIRA involved in CABIN1 interaction could be distinct from the one required for HIRA homooligomerization. Secondary structure prediction of the C-terminal half of the HIRA protein suggested three distinct regions, a β-strand domain (aa 661–872) in which we delineated critical amino acids for HIRA homooligomerization (W799 and D800), a loop (aa 873–904) and a α-helical domain (aa 905–1017) (Fig. 3a; Supplementary Fig. 1a). We hypothesized that either the loop or the α-helical domain could contribute to CABIN1 binding. To test this hypothesis, we generated additional HIRA-YFP mutant constructs (Fig. 3a) and, after U2OS cells transfection and preparation of extracts, we performed anti-GFP immunoprecipitations (Fig. 3b). The HIRA(1–918) mutant lacking the extreme C-terminal α-helical domain was able to co-immunoprecipitate CABIN1 similarly to the wt protein. In contrast, both the HIRA(Δ862–962) and HIRA(Δ873–904) mutants exhibited a reduced amount of co-immunoprecipitated CABIN1 as compared to the wt protein, indicating that the loop region contributes to CABIN1 binding. Notably, HIRA(Δ862–962) was found to co-immunoprecipitate HIRA-HA similarly to the wt protein (Fig. 1c) showing that the domain of HIRA involved in CABIN1 interaction is distinct from the domain required for HIRA homooligomerization. To identify potential amino acids critical for CABIN1 interaction in the loop, we generated amino acids mutants HIRA(FRL870-871-874AAA), HIRA(R892A-N895A), and HIRA(FRLRN870-871-874-892-895AAAAA) by changing the indicated residues to alanines (Supplementary Fig. 1b; Supplementary Fig. 5a). Both HIRA(FRL870-871-874AAA) and HIRA(FRLRN870-871-874-892-895AAAAA) mutants exhibited a decrease in the amount of co-immunoprecipitated CABIN1, as compared to wt and HIRA(R892A-N895A) proteins, suggesting that the HIRA amino acids F870, R871, and/or L874 within the loop region are required for efficient binding to CABIN1 (Supplementary Fig. 5b).

We generated a HeLa cell line knockout for HIRA by the CRISPR-Cas9 technology. Western blot analysis showed that the absence of HIRA leads to the decrease of both CABIN1 and UBN1 (Fig. 3c, left). We transfected these HIRA KO cells with HIRA-YFP wt (W799A–D800A) and (Δ873–904) and evaluated the recovery of CABIN1 and UBN1 by western blot analysis. Expression of each of these three YFP-tagged proteins induced an increase of UBN1 protein as compared to the YFP control whereas only the wt protein led to an increase of CABIN1 (Fig. 3c, right). This argues that both the homooligomerization (W799A–D800A) and loop deletion (Δ873–904) HIRA mutants were unable to induce a partial recovery of CABIN1 as compared to HIRA wt, possibly due to their defect in CABIN1 binding, as suggested by our co-immunoprecipitation data.

We used Superose 6 fractionation of recombinant proteins to analyze the direct interaction between the loop region of HIRA and CABIN1 (Fig. 3d). As compared to CABIN1 FL alone, mixing HIRA(661–1017) and CABIN1 FL resulted in an earlier elution peak containing both proteins indicative of a protein complex. Mixing CABIN1 FL and the loop deletion mutant HIRA(661–1017) Δ873–904 resulted in a reduced peak corresponding to protein complex (Fig. 3d; Supplementary Fig. 5d) confirming in vitro that the loop region plays a role in the interaction of HIRA with CABIN1. We found a similar reduction in the protein complex peak, although less pronounced, with HIRA(661–1017 + FRL870-871-874AAA) (Supplementary Fig. 5e,f). Moreover, mixing CABIN1 FL with either the β-strand (661–872) or the loop + α-helical (873–1017) domains of HIRA did not show a detectable protein complex peak (Supplementary Fig. 5e,f). This indicates that, although the loop participates in CABIN1 interaction, it is not sufficient on its own and also requires the β-strand domain involved in HIRA homooligomerization. Furthermore, while this loop deletion mutant HIRA(661–1017) Δ873–904 is deficient for CABIN1 interaction we showed that it can form a homotrimer as the wt protein by sedimentation equilibrium analysis (Fig. 3e; Supplementary Fig. 5c), arguing that the regions of HIRA involved in CABIN1 interaction and homooligomerization are distinct. These findings indicate that CABIN1 binding to HIRA requires HIRA homooligomerization and involves the loop domain (873–904).

### Crystal structure of HIRA(644–1017) reveals a homotrimer

**Fig. 4:**
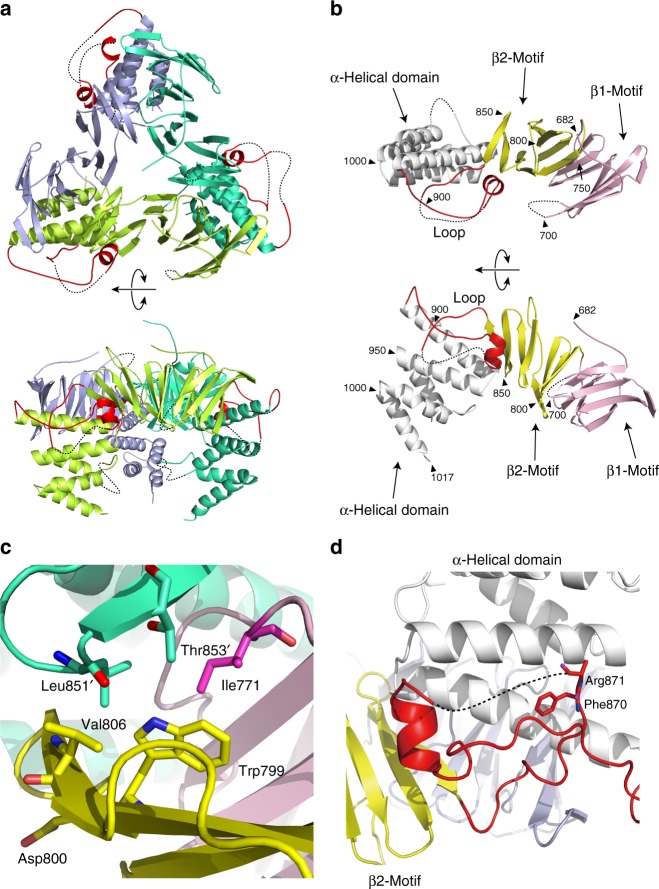
Crystal structure of HIRA(644–1017) reveals a homotrimer. **a** Overall structure of homotrimeric HIRA(644–1017). Subunits A, B and C are shown in light green, emerald green, and blue, respectively. The loop regions (860–907) are shown in red. **b** Structure of the HIRA monomer. The β1 and β2 motifs are shown in pink and yellow, respectively. The α-helical domain and the loop region are shown in white and red, respectively. Black triangles indicate residue numbers of HIRA(644–1017). **c** Trp799 located at the interface between subunits A and B (emerald green). **d** The CABIN1-binding loop shown in red and highlighting Phe870 and Arg871. The side chain of Arg871 was not visible in the crystal structure. Residues in the α-helical domain are colored white

To determine the molecular basis for HIRA homooligomerization, we determined the crystal structure of the C-terminal half of HIRA (aa 644–1017) at 2.4 Å resolution (Table 1; Supplementary Fig. 6). The asymmetric unit of the crystal contained a homotrimer of HIRA(644–1017) (Fig. 4). This is consistent with our in vitro studies and analysis by size-exclusion chromatography with in-line multi-angle light scattering (SEC-MALS) (Supplementary Fig. 6c; Supplementary Methods), yielding a molecular weight of HIRA(644–1017) of 111,400 Da ± 2.3 % in solution. The observed average value for inter-subunit buried surface area of the homotrimer is ∼1440 Å^2^,consistent with its high stability. Structures of the three subunits are nearly identical with an averaged root mean square deviation (RMSD) of about 0.8 Å with structural differences largely restricted to loop regions. We confirmed the existence of three distinct domains as previously predicted (Supplementary Fig. 1a) and refined the domain boundaries, β-strand (residues 691–859), loop (residues 860–907), and α-helical (residues 908–1017) (Fig. 4a). N-terminal residues 644–674 were not visible in the crystal structure, suggesting that this region of HIRA(644–1017) is highly flexible. The trimer is arranged in a ring of two layers, composed of β-strands and α-helices, respectively. The β-strand domain is composed of two β-sandwich motifs, β1 (residues 682–774) and β2 (residues 775–860) motifs (Fig. 4b). The β1 motif of one subunit interacts with the β2 motif and α-helical domain of an adjacent subunit in the homotrimer.

**Table 1:** Data collection, phasing and refinement statistics for the X-ray structure of HIRA(644–1017)

	Se-SAD	MR-native SAD	Native
*Data collection*			
Space group	*C*2	*C*2	*C*2
Cell dimensions			
* a*, *b*, *c* (Å)	149.41, 86.85, 98.11	149.65, 86.61, 99.03	149.99, 86.80, 99.15
* α*, *β*, γ (°)	90.00, 101.67, 90.00	90.00, 102.51, 90.00	90.00, 102.49, 90.00
Wavelength	0.9790	1.9000	0.9800
Resolution (Å)	20.00–2.55 (2.62–2.55)^a^	20.00–2.80 (2.87–2.80)	20.00–2.45 (2.51–2.45)
*R*_merge_	0.05 (0.46)	0.07 (0.46)	0.04 (0.50)
*I*/*σI*	14.97 (2.88)	30.35 (6.55)	19.40 (2.86)
Completeness (%)	98.3 (99.0)	99.6 (100.2)	99.5 (99.8)
Redundancy	3.55 (3.62)	13.58 (13.33)	3.48 (3.50)
*Refinement*			
Resolution (Å)			20.00–2.45
No. of reflections			89,387
*R*_work_/*R*_free_			0.2017/0.2562
No. of atoms			
Protein			6807
Ion			15
Water			16
*B*-factors			
Protein			59.5
Ion			65.4
Water			52.0
R.m.s deviations			
Bond lengths (Å)			0.008
Bond angles (°)			1.026

^a^Values in parentheses are for highest-resolution shell.

Trp799 and/or Asp800, which were identified as critical residues for homotrimer formation (Fig. 1c, d), are located in the β2 motif. While Asp800 does not interact with an adjacent subunit, Trp799 is located at the interface of the two subunits and forms a hydrophobic patch with Ile771 and Val806. This hydrophobic patch interacts with Leu851 and Thr853 of an adjacent subunit (Fig. 4c; Supplementary Fig. 6e). Substitution of Trp799 apparently significantly reduces the hydrophobic interaction between the subunits. In addition, HIRA(Δ862–962), which lacks the loop and about a half of the α-helical domain, still forms a homotrimer (Fig. 1c). These results suggested that interaction between the α-helical and β-strand domains is not essential for homotrimer formation. The residues suggested to be required for CABIN1 interaction, Phe870, Arg871, and Leu874 (Supplementary Fig. 5a,b,e,f), are located in the loop region (Fig. 4d) and Phe870 and Arg871 seem to form a CABIN1-binding surface, despite the fact that most of the loop region, including Leu874, are not visible in the crystal structure.

**Fig. 5:**
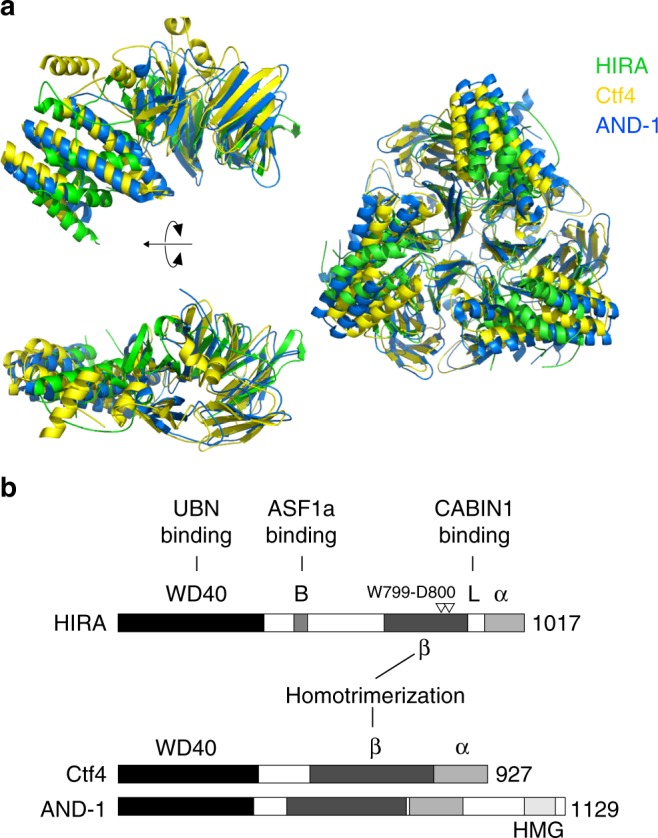
Homotrimeric HIRA(644–1017) exhibits structural homology with the homotrimeric replisome factor Ctf4/AND-1. **a** (Left) Superpositions of the HIRA(644–1017) monomer (green) onto Ctf4 (yellow) and AND-1 (blue) monomers. These superpositions with Ctf4 and AND-1 give RMSDs values of 3.9 and 4.1 Å for 197 and 204 Cα atoms, respectively. (Right) Superpositions of the HIRA(644–1017) trimer onto Ctf4 (RMSD = 4.6 Å with 620 Cα atoms) and AND-1 (RMSD = 4.2 Å with 629 Cα atoms) trimers. These superpositions were performed using the program SUPERPOSE in CCP4^49^. **b** Scheme of the predicted secondary structures of human HIRA, yeast Ctf4, and its human counterpart AND-1. They exhibit similarities, with a β-strand domain involved in the homotrimerization of the proteins, an α-helical domain in the C-terminus, and a WD40-repeat region in the N-terminus. The amino acids W799–D800 found critical for HIRA homotrimerization are indicated

Analysis with the DALI server (http://ekhidna2.biocenter.helsinki.fi/dali/) revealed that the overall structure of homotrimeric HIRA(644–1017) is similar to the yeast replisome factor Ctf4 and its human counterpart AND-1^28,29^ (Fig. 5a). Each of these homotrimeric structures contain β-strand and α-helical layers, and the relative arrangement of the two domains are similar. Despite these similarities, HIRA(644–1017) lacks a six-bladed β-propeller structure of the β-strand domain found in the other protein complexes. The loop domain (873–904) of HIRA(644–1017) was present close to the missing β-propeller. Moreover, HIRA and Ctf4/AND-1 proteins also share an overall secondary structure similarity, notably with a WD40 repeat domain in their N-terminal half (Fig. 5b). The crystal structure of HIRA(644–1017) provides evidence that the HIRA subunit forms a homotrimer with intriguing structural homology with the replisome factor Ctf4/AND-1.

### HIRA homotrimerization is required for its activity

**Fig. 6:**
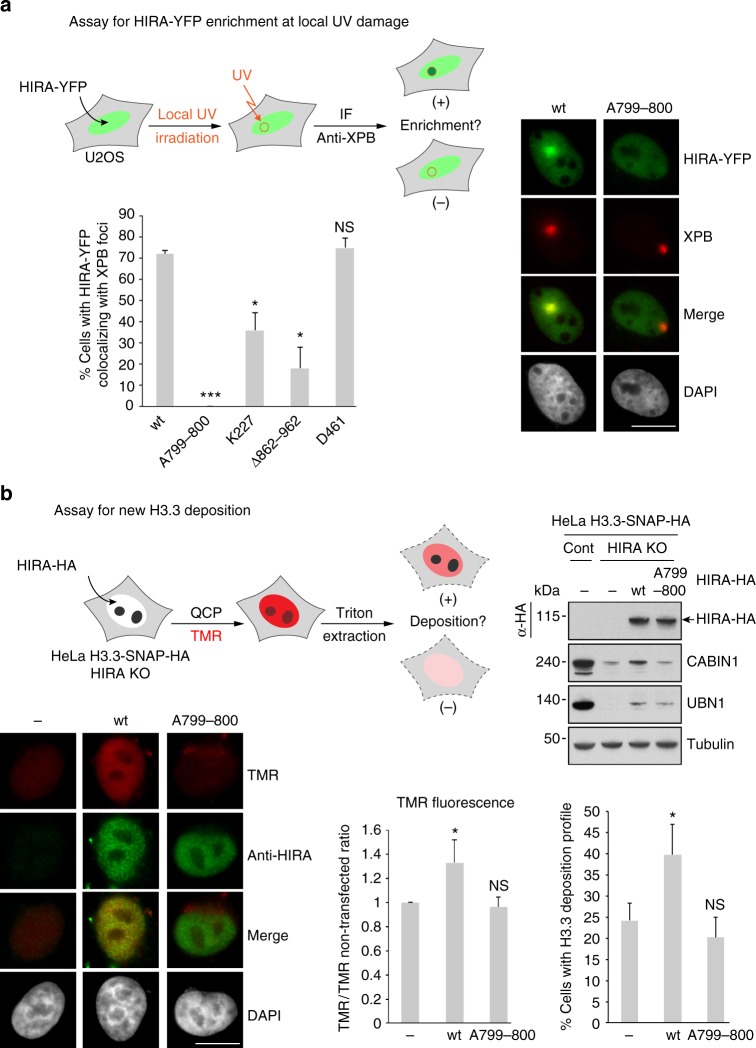
Homotrimerization of HIRA is critical for its enrichment at UV damaged sites and for new H3.3 deposition. **a** (Left, top) Scheme of the experimental assay for HIRA-YFP enrichment at local UV damage in U2OS cells. (Left, bottom) The graph shows the percentage of U2OS cells with HIRA-YFP protein colocalizing with XPB foci. Error bars represent SD from three independent experiments. Statistical analysis using a *t*-test was performed with Prism 7 software (ns: nonsignificant, *p < 0.05, ***p < 0.001). (Right) Images of representative U2OS cells irradiated locally with UV exhibiting an enrichment of HIRA wt or an absence of enrichment of HIRA (W799A–D800A) at one damage site identified by anti-XPB immunofluorescence. Scale bar 5 μm. **b** (Top, left) Scheme of the experimental assay for new H3.3 deposition by a Quench-Chase-Pulse experiment (QCP) in HeLa H3.3-SNAP-HA HIRA KO cells transfected with HIRA-HA constructs. (Top, right) Western blot analysis showing the protein expression of exogeneous HIRA-HA wt and (W799A–D800A) mutant. (Bottom, left) Images of cells subjected to QCP: representative images for a cell non-transfected or transfected with HIRA-HA (W799A–D800A) mutant and a high TMR positive representative image for a cell transfected with HIRA-HA wt. (Bottom, right). One graph shows the fluorescence intensity ratio TMR/TMR non-transfected. The other graph gives the percentage of cells exhibiting a positive profile for H3.3 deposition. Error bars represent the SD from three independent experiments. Statistical analysis using a *t*-test was performed with Prism 7 software (ns: nonsignificant, *p < 0.05)

We previously found that the HIRA complex is enriched at UV damage sites and this is necessary for efficient transcription re-start after repair^13^. To address whether the homotrimerization of HIRA plays a role in its biological activity, we aimed to analyze the enrichment of HIRA-YFP mutant proteins at DNA damage sites (Scheme Fig. 6a). We first verified the nuclear localization of both wt and mutant HIRA-YFP proteins without UV irradiation (Supplementary Fig. 7a). Notably, in contrast to the other YFP-HIRA proteins, an AT-hook mutant was not properly localized in the nuclei of expressing cells. This AT-hook motif, conserved in HIRA proteins of higher eukaryotes (Supplementary Fig. 1b)^27^, has been previously identified as part of a potential basic bipartite nuclear localization signal (NLS)^4^ and we found here that it is indeed required for the nuclear localization of the HIRA protein. To evaluate the capacity of the different HIRA-YFP proteins to be enriched at UV damage sites 30 min after local UV irradiation (Fig. 6a), we determined the percentage of cells with HIRA-YFP protein colocalizing with the repair protein Xeroderma pigmentosum complementation group B (XPB) foci (Fig. 6a graph). We found 72% and 75% of cells with HIRA-wt and HIRA(I461D) (mutant for ASF1a interaction) colocalizing with XPB foci, respectively, indicating that ASF1a binding is not necessary for HIRA to be enriched at damage sites. In contrast, we observed only 36%, 18% and 0% of cells with HIRA(R227K) (mutant for UBN1 interaction), HIRA(Δ862–962) (mutant for CABIN1 interaction) and HIRA(W799A–D800A) (homotrimerization mutant) colocalizing with XPB foci, respectively. A representative colocalization of HIRA-wt and absence of colocalization of HIRA(W799A–D800A) with XPB at a damage site are shown (Fig. 6a, right). These results indicate that UBN1 and CABIN1 bindings are required for a fully efficient enrichment of HIRA at UV damage sites and more dramatically that the lack of HIRA homotrimerization completely prevents its enrichment.

To address further whether the homootrimerization of HIRA plays a role in the functional activity of the complex, we aimed to analyze its impact on the deposition of the histone variant H3.3. We first verified by anti-HA co-immunoprecipitation that a HIRA homooligomerization mutant interacts with H3.3-SNAP-HA, as expected since this mutant still interacts with UBN1, which is the subunit within the complex that confers H3.3 binding^14^ (Supplementary Fig. 4c). We then analyzed the capacity of HIRA wt and HIRA(W799A–D800A) trimerization mutant to rescue the deposition of new H3.3 histone variant in HIRA knockout cells (Scheme Fig. 6b). We generated a HIRA KO cell line from the previously described HeLa H3.3-SNAP-HA cell line^5^ by the CRISPR-Cas9 technology. The lack of HIRA expression, accompanied by the decrease of both CABIN1 and UBN1, was verified by western blot analysis (Supplementary Fig. 7b). We performed Quench-Chase-Pulse experiments that allow the visualization of newly synthesized H3.3 in cells via the SNAP-tag. By comparing H3.3-SNAP-HA HIRA KO and control cells, we found that the deposition of newly synthesized H3.3 is affected in the absence of HIRA, as previously described in siHIRA treated cells^5^ (Supplementary Fig. 7c,d). We transfected HIRA KO cells with either HIRA-HA wt or (W799A–D800A) mutant and analyzed new H3.3 deposition. We observed a significant increase of new H3.3 deposition in cells expressing HIRA-HA wt but not in cells expressing HIRA-HA (W799A–D800A) mutant (Fig. 6b). This result was obtained both by quantifying the TMR fluorescence intensity and by counting the cells exhibiting a H3.3 deposition profile (graphs Fig. 6b). We show representative cells with low TMR signal for both non-transfected and HIRA-HA (W799A–D800A) transfected cells and a high TMR representative cell for HIRA-HA wt transfected cells (Fig. 6b, bottom left). The comparable expression of both HIRA-HA wt and mutant proteins in transfected HIRA KO cells was verified by western blot (Fig. 6b, top right). However, the low increase of both CABIN1 and UBN1 in HIRA-HA wt transfected HIRA KO cells as compared to their expression level in control cells may explain the observation of only partial rescue of H3.3 deposition when compared to its efficiency in control cells (graphs Supplementary Fig. 7d). From these results, we conclude that a homotrimerization mutant is deficient both in its capacity to be enriched at UV damage sites and to deposit new H3.3, arguing that homotrimerization of the HIRA subunit is crucial for the functional activity of the HIRA complex.

## Discussion

**Fig. 7:**
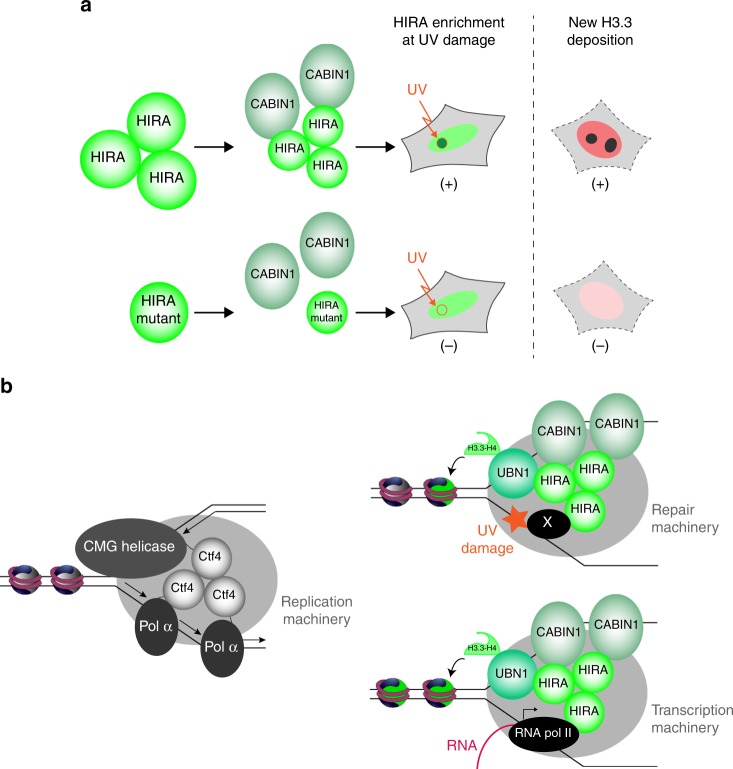
HIRA homotrimerization: its role in HIRA activity and structural similarity with Ctf4/AND-1. **a** Scheme of HIRA homotrimerization that is required for CABIN1 interaction, HIRA enrichment at UV damage site and deposition of new H3.3 histone variant. A HIRA homotrimerization mutant cannot interact with CABIN1, is not enriched at UV damage sites and cannot deposit new H3.3. **b** Models for Ctf4 and HIRA homotrimers functions. (Left) Within the replisome, Ctf4 homotrimer functions as a bridge coupling two molecules of DNA polymerase α on the lagging strand to one CMG (Cdc45-MCM-GINS) helicase on the leading strand^28^. (Right) A HIRA homotrimer might act as a platform interacting with components of transcription or repair machineries in conjunction with binding to ssDNA at DNA bubble structures, allowing H3.3 deposition at these sites. HIRA may interact with RNA pol II machinery in the context of transcription as previously proposed^5^, while it may bind to a non-identified repair protein (X) in the context of DNA damage. Two CABIN1 molecules are shown, as a HIRA homotrimer was found to interact with two CABIN1 molecules in vitro (Fig. 1e). The number of UBN1 molecules associated with a HIRA homotrimer being not known, only one molecule is represented

Our study revealed that the HIRA subunit homooligomerizes in cells and that the most stable form is a homotrimer in vitro. We showed that HIRA homotrimerization is necessary for its interaction with CABIN1, is absolutely required for its enrichment at UV damage sites and critical for efficient new H3.3 deposition (Fig. 7a). The crystal structure highlights that the previously described C domain of HIRA can be divided into three different structural regions: β-strand, loop, and α-helical domains. While we find that the β-strand region is essential for homotrimerization, the loop region appears to be important for direct interaction with CABIN1. Importantly, the β-strand domain is also required for CABIN1 interaction arguing that CABIN1 is able to bind only to the homotrimeric form of HIRA. The fact that a recombinant trimerization mutant protein, HIRA(661–1017 + W799A-D800A), elutes in the void volume of a gel filtration column (Supplementary Fig. 2c) argues that the HIRA(661–1017) as a monomer is likely unfolded in vitro. We hypothesize that the homotrimeric structure of HIRA generates a conformational state allowing for its interaction with CABIN1 that occurs primarily through the loop region. Although it is possible that CABIN1 binding to HIRA could contribute to HIRA homotrimerization, our observation that CABIN1 depletion by siRNA still results in HIRA self-association in cells argues that HIRA can homotrimerize independently of CABIN1 binding.

Interestingly, we showed that the HIRA–CABIN1 complex likely contains three HIRA and two CABIN1 molecules in vitro (Fig. 1e). There are at least two possibilities that could explain the unbalanced stoichiometry of the HIRA–CABIN1 complex on the basis of structural features of HIRA and CABIN1 (Supplementary Fig. 8a). Since CABIN1 is a large molecule of 2200 amino-acid residues, the homotrimer of HIRA(644–1017) could be too small to accommodate three CABIN1 molecules. It is also possible that upon CABIN1 binding, the HIRA trimer undergoes a structural change that disrupts the three-fold symmetry of the HIRA trimer to better accommodate two molecules of CABIN1. Both possibilities are not mutually exclusive and a resolution of the molecular basis for understanding the 3:2 HIRA–CABIN1 stoichiometry must await a complete structure of the whole complex. The stoichiometry of the intact HIRA complex including UBN1, which is the subunit that binds directly to H3.3^14^, is not yet known. We previously reported that the WD40 repeat domain of HIRA and the NHRD (N-terminal to the HRD) of UBN1 form a 1:1 complex^30^ but how many UBN1 molecules are associated with the homotrimeric HIRA remains to be determined. Indeed, this is of particular interest since several H3 histone chaperones have been shown to homodimerize leading to the assumption that this homodimerization allows for the simultaneous deposition of two H3–H4 dimers to form a nucleosome^31^. Whether the HIRA complex, with the homotrimeric structure of the HIRA subunit, keeps this mode of histone deposition will be important to determine. It may also be that the three subunits of HIRA are not equivalent and while two HIRA protomers could behave in a manner resembling known histone chaperones, the third HIRA protomer could provide an additional functional property.

We found that a homotrimerization mutant does not accumulate at local UV damage sites (Fig. 6a). This indicates that homotrimerization is absolutely required for HIRA to accumulate and argues that HIRA as a monomer, that does not bind to CABIN1 but still interact with both ASF1a and UBN1, cannot be enriched at DNA damage. Notably, we previously found that depletion of CABIN1 reduces the accumulation of the HIRA subunit at UV damage sites suggesting that CABIN1 participates in the targeting and/or stabilization of HIRA at these sites^13^. Moreover, we showed that a HIRA homotrimerization mutant does not recover the deposition of new H3.3 in HIRA KO cells while HIRA wt can partially rescue. A recent study, using cell free extracts from *Xenopus* eggs, showed that while the N-terminal half of HIRA (containing both UBN and ASF1a binding domains) is sufficient to perform nucleosome assembly in vitro, the C-terminal half of HIRA (containing both the homotrimerization and CABIN1-binding domains) is involved in chromatin binding^32^. It will be interesting to investigate whether the deficiency of a HIRA homotrimerization mutant in rescuing new H3.3 deposition in HIRA KO cells is only due to the defect in its chromatin targeting or if HIRA homotrimerization is also required in cells for the H3.3 deposition process per se.

The functional significance of homotrimetric HIRA could be deduced from its overall structural similarity to Ctf4/AND-1 (Fig. 5), which functions as a scaffold protein in the replisome. Within the replisome, Ctf4 functions as a bridge coupling two molecules of DNA polymerase α on the lagging strand to one CMG (Cdc45-MCM-GINS) helicase on the leading strand (Fig. 7b left). It is proposed that this homotrimeric structure allows Ctf4 to connect multiple accessory factors to the replisome^28,33^. Similarly, the Ctf4 ortholog in higher eukaryotes, AND-1, acts as a component of the replisome to regulate DNA replication and S phase progression^34,35^ and forms a homotrimer^29^. Intriguingly, AND-1 has also been reported to interact with the centromeric H3, CenH3^CENP-A^, and to participate together with the chaperone Holliday Junction Recognition Protein (HJURP) in its deposition process onto DNA^36^. AND-1 exhibits DNA-binding capacity and we previously showed that the HIRA complex is able to bind double-stranded (ds) DNA^5^. However, while a High-Mobility Group (HMG) DNA binding motif is present in the C-terminus of AND-1^37^, no DNA binding motif has been identified so far in HIRA. AND-1 can bind both ds and single-stranded (ss) DNA^29,38^ and interestingly our preliminary work indicates that the HIRA complex from extracts can also bind ssDNA (Supplementary Fig. 8b). Whether this exploits RPA, a ssDNA binding protein complex which has been proposed to recruit the HIRA complex at promoters and enhancers^21^, remains to be explored. Our findings, in light of its homology with Ctf4/AND-1, allow us to propose models for H3.3 deposition through the HIRA complex (Fig. 7b right). The homotrimeric HIRA might act as a platform for multiple interactions at sites of transcription or repair. HIRA interaction with component(s) of transcription (already described^5^) or repair machineries in conjunction with binding to ssDNA, present at these DNA bubble structures, may help to recruit/maintain the HIRA complex for H3.3 deposition.

In conclusion, our study, by revealing the homotrimerization of the HIRA subunit, provides a structural view of the HIRA complex which is of critical importance for elucidating its mechanistic activity in the process of H3.3 deposition and potentially in additional functions.

## Methods

### Generation of expression plasmids

The plasmids encoding GST-HIRA(661–1017), GST-HIRA(661–872), GST-HIRA(905–1017), and GST-HIRA(873–1017) were generated by PCR amplification from a FL human HIRA construct^15,30^ and ligation into the EcoRI/XhoI sites of a custom engineered pRSFduet-1 (Novagen) *Escherichia coli* expression vector carrying a N-terminal GST tag that is removable by TEV protease cleavage. The plasmids encoding GST-HIRA(661–1017, Δ873–904) and GST-HIRA(661–1017) F870A-R871A-L874A were generated by site-directed mutagenesis^39^. The plasmid encoding FL His-CABIN1 for expression in Sf9 insect cells using the baculovirus system was generated by ligation into the EcoRI/SphI sites of the pFastBac™ HTA vector (Fisher Scientific).

The plasmids encoding HIRA-YFP wt and deletion mutants were generated by PCR amplification from a FL human HIRA construct and cloned into the pEYFP-N1 plasmid (Clontech). The plasmid encoding HIRA-HA wt was previously described^13^. The amino acids mutants in HIRA-YFP and HIRA-HA plasmids were generated by directed mutagenesis (QuickChange II, Site-Directed Mutagenesis Kit, Agilent Technologies).

The expression vector for crystallographic analysis contains residues 644–1017 of human HIRA. The *hira*(644–1017) gene was amplified by PCR using primers, 5′-GAGATATACATATGGGGCGGCCTCGGAAGGACTC-3′ (an NdeI site is underlined) and 5′-CAGCTCGACATCCTGAGGGACAAGCTCGAGGGCTCTTCC-3′ (XhoI and SapI sites are underlined) with cDNA of human HIRA from Mammalian Gene Collection clones (GE Healthcare) as a template. The amplified DNA fragment was cloned into the NdeI and XhoI sites of pTXB1 (New England BioLabs), resulting in pTXB1-*hira*(644–1017) (pYHD1).

### HeLa and HeLa H3.3-SNAP-HA cell lines knockout for HIRA

Pseudotyped lentiviral particles were generated with LentiCRISPR v2 vector by cotransfection with psPAX2 and pMD2.G vectors at a ratio of 4:3:1 into Lenti-X-293T cells (Clontech) using JetPRIME (Polyplus). Cell infection of HeLa B (from Sandrine Middendorf) and HeLa H3.3-SNAP-HA (from Lars Jansen)^5^ was carried out in the presence of 8 μg/ml polybrene (Sigma). Twenty-four hours later, transduced cells were selected with 1 μg/ml puromycin (purchased from Life Technologies) and maintained in the presence of drug for 2 weeks. Polyclonal population of cells was used as a crGFP control. To obtain HIRA KO cell lines, single cell clones were obtained by limiting dilution. The presence of rearrangements in the *HIRA* gene was confirmed by Sanger sequencing. gRNA sequence targeting GFP (5′- GAGCTGGACGGCGACGTAAA-3′) were cloned into the lentiCRISPR v2 plasmid^40^. LentiCRISPR v2 plasmids containing gRNA sequences against human *HIRA* (5′-TACCTAAGTGATTGTCCATC-3′) was obtained from Genescript. This sequence targets the exon 3–intron boundary of *HIRA*.

### Cell Transfection and extract

Human U2OS (from Jiri Bartek) and HeLa cells were transfected using lipofectamine 2000 and lipofectamine RNAIMAX (Invitrogen) for plasmids and siRNAs, respectively. Small interfering RNAs (siRNA) were purchased from Dharmacon. We used ON-TARGETplus J-012454-09 (CABIN1) and D-001810-01 (non-targeting control).

Nuclear extracts from U2OS or HeLa cells were obtained as previously described except that 300 mM NaCl was used^41^.

### UV irradiation and immunofluorescence

U2OS cells were subjected to local UV-C (254 nm) irradiation (300 J/m^2^) 8 h post-transfection using a low-pressure mercury lamp and 5 μm pore filters (Millipore)^13^.

For immunofluorescence, cells were fixed in 2% paraformaldehyde, permeabilized 5 min with 0.2% Triton in PBS and blocked with BSA (5% in PBS plus 0.1% Tween) before incubation with primary and secondary antibodies and DAPI staining. Coverslips were mounted in Vectashield medium. We used a Zeiss Z1 microscope (40 and 63× objectives).

### Immunoprecipitation and western blotting

GFP immunoprecipitations were carried out overnight at 4 °C with GFP-Trap coupled to agarose beads (ChromoTek) in the presence of 150 mM NaCl and 0.5% Nonidet-P40 substitute. For the washes 300 mM NaCl was used.

For western blot analysis, extracts or immunoprecipitated proteins were run on NuPAGE bis–tris 4–12% gels in MOPS buffer (Invitrogen) and transferred to nitrocellulose membrane (Protran). Primary antibodies were detected using horse-radish-peroxidase-conjugated secondary antibodies (Jackson ImmunoResearch Laboratories) and SuperSignal enhanced chemiluminescent detection kit (Pierce). The molecular weights indicated on the western blots correspond to the PageRuler Prestained Protein Ladder (26616, Thermo Scientific) except the 240 kDa which corresponds to CABIN1 molecular weight. Uncropped scans of the blots shown in the main figures are provided in supplementary Figs. 9, 10 and 11.

### New H3.3-SNAP labeling and fluorescence quantification

We performed the labeling of new H3.3-SNAP by the Quench-Chase-Pulse experiment^5,42^. We added to cell medium at 37 °C 10 μM of SNAP-Cell Block (Biolabs) during 30 min to quench SNAP-tag activity (Quench), we incubated the cells for 2 h in complete medium at 37 °C for expression of new H3.3-SNAP (Chase) and finally added to cell medium at 37 °C 2 μM of SNAP-Cell TMR-Star (Biolabs) during 20 min to label newly synthesized H3.3-SNAP (Pulse). After this in vivo labeling, the cells were triton pre-extracted and used for SNAP labeling visualization by microscopy. To quantify fluorescence intensity (TMR signal) in the acquired images we used the ImageJ software^5,42^. We performed three independent experiments and quantified the fluorescence intensity of at least 400 nuclei for each condition. Alternatively, from the same acquired images we counted the number of cells with positive H3.3 deposition profile.

### Antibodies

Antibodies were used at the following dilutions: anti-HIRA mouse monoclonal (WC119)^43^ western and IF 1:100; anti-CABIN1 rabbit polyclonal (ab3349, or 76600, Abcam) western 1:1000; anti-UBN1 rabbit polyclonal (ab101282, Abcam) western 1:1000; anti-HA epitope rat monoclonal (Roche) western 1:1000; anti-ASF1a rabbit polyclonal (#2990, Cell Signaling) western 1:1000; anti-GFP rabbit polyclonal (platform Curie) western 1:1000 or mouse monoclonal (11 814 460 001, Roche) western 1:1000; anti-XPB (or TFIIH p89) rabbit polyclonal (sc-293, Santa Cruz Biotechnology) IF 1:300; anti-γtubulin mouse monoclonal (T5326, Sigma); anti-p53 rabbit polyclonal (sc-6243, Santa Cruz Biotechnology) western 1:500; anti-DAXX rabbit monoclonal (#4533, Cell Signaling) western 1:500; anti-p150 CAF-1 mouse monoclonal (ab7655, Abcam) western 1:500; anti-p60 CAF-1 rabbit polyclonal western 1:1000 ^44^.

### Expression and purification of recombinant proteins

For biochemical analysis,. GST-HIRA(661–1017), GST-HIRA(661–872), GST-HIRA(905–1017), GST-HIRA(873–1017), GST-HIRA(661–1017, D873–904), and GST-HIRA(661–1017) F870A-R871A-L874A were produced in BL21-Gold(DE3) *E. coli* cells (Agilent) induced with 0.8 mM IPTG and expressed overnight at 18 °C. Cells were re-suspended in 1XPBS supplemented with 5 mM BME and lysed by sonication. Lysate was clarified by centrifugation and supernatant was subjected to GST affinity chromatography. GST fusion proteins were then subjected to on-column cleavage overnight though incubation with TEV protease. Proteins were then eluted into buffer containing 20 mM Tris pH 8.0, 50 mM NaCl, and 5 mM BME and subjected to ion exchange chromatography using a HiTrap Q column (GE Healthcare). Following ion exchange, proteins were concentrated using a spin concentrator (Millipore) and loaded onto a Superdex 200 10/300 GL or Superose 6 10/300 GL (GE Healthcare) column for further purification by size-exclusion in a buffer containing 20 mM Tris pH 8.0, 300 mM NaCl, and 1 mM TCEP. Full-length His-CABIN1 was expressed in Sf9 inscet cells using the baculovirus system^19^. Sf9 cells in which CABIN1 was expressed were re-suspended in buffer containing 20 mM Tris pH 8.0, 500 mM NaCl, 5 mM BME, and 10 mM imidazole and lysed with sonication. Lysate was clarified with centrifugation and the supernatant was subjected to nickel affinity chromatography to isolate the His tagged protein. Protein was eluted with 200 mM Imidazole and dialyzed overnight into buffer with 20 mM Tris pH 8.0, 300 mM NaCl, 1 mM TCEP. Protein was then concentrated using proteins were concentrated to a volumn of 500 µL using a spin concentrator (Millipore) and resolved on a Superose 6 10/300 GL column in a buffer with 20 mM Tris pH 8.0, 300 mM NaCl, 1 mM TCEP.

For crystallographic analysis, *E*. *coli* BL21(DE3) was transformed with the expression vector pYHD1 and cultured in LB medium containing 0.2% (w/v) glucose, 0.2% (v/v) glycerol and 100 μg/ml carbenicillin at 310 K. The expression of HIRA(644–1017) was induced by adding 0.5 mM isopropyl-β-d-thiogalactopyranosid (IPTG) when the culture reached an OD_600_ of 0.7. After further incubation for 24 h at 298 K, the cells were harvested, washed twice with 20 mM Tris–HCl (pH 8.5), 10% (v/v) glycerol, and 50 mM NaCl (buffer A), and re-suspended in the same buffer.

For Selenomethionine(Se-Met)-labeled protein expression, *E*. *coli* BL834(DE3) was transformed with the expression vector pYHD1 and cultured in Lemaster medium^45^ containing 100 μg/ml carbenicillin and 50 μg/ml seleno-l-methionine at 310 K. The expression of Se-Met-labeled HIRA(644–1017) (Se-Met HIRA(644–1017)) was induced by adding 0.5 mM IPTG when the culture reached an OD_600_ of 0.7. After further incubation for 24 h at 298 K the cells were harvested, washed twice with buffer A and re-suspended in the same buffer.

The expressed HIRA(644–1017) or Se-Met HIRA(644–1017) was purified as follows. The cells were incubated with buffer A containing 1 mM magnesium chloride, 0.4 mM ATP, 0.02% (w/v) 3-[(3-cholamidopropyl)-dimethylammonio]-propanesulfonate (CHAPS), and 1 U/ml Benozase nuclease (Merck) at 277 K for 30 min. The cells were lysed by EmulsiFlex C5 (Avestin) and then further disrupted by sonication using a VP-30S (TAITEC). Cell debris and large particles were removed by centrifugation at 15,000×*g* for 15 min. The extract was bound to chitin beads (New England BioLabs), which has been previously equilibrated in buffer A, on a rotary shaker for 1 h at 277 K. Then, the mixture was loaded onto a Pierce centrifuge column (Thermo Fisher Scientific) and washed with 20 mM Tris–HCl (pH 8.5), 10% (v/v) glycerol, and 300 mM NaCl (buffer B) for reduction of the nonspecific binding of proteins. On-column Intein cleavage was induced by adding 20 mM Tris–HCl (pH 8.5), 10% (v/v) glycerol, 500 mM NaCl, and 50 mM DTT (buffer C) at 293 K. The cleavage reaction of HIRA(644–1017) or Se-Met HIRA(644–1017) was carried out for 40 h. The eluted protein was further purified by size-exclusion chromatography using a HiLoad 16/600 superdex 200 pg column (GE Healthcare) equilibrated with 20 mM HEPES–NaOH (pH 6.8), 10% (v/v) glycerol, 500 mM NaCl, and 2 mM DTT. The purified protein was concentrated using Amicon Ultra-15 (Merck). The concentration of protein was measured by absorbance at 280 nm (*ε*_HIRA(644–1017)_ = 9.9 for 1% (w/v) solution). After the Intein cleavage, HIRA(644–1017) contains five additional amino acid residues derived from pTXB1 sequence (Leu-Glu-Gly-Ser-Ser) at the C-terminus. Images of Coomassie-stained gels were obtained using Odyssey imaging system (LI-COR Biosciences).

### Size-exclusion chromatography

To monitor formation of complexes containing recombinant HIRA C-terminal fragments and recombinant full-length CABIN1, HIRA was incubated in a five-fold molar excess with CABIN1, ~100 μM HIRA incubated with ~20 μM CABIN1 at a volume of 500 μl. The mixed proteins were allowed to incubate at 4 °C for 30 min for complex formation prior to resolving on a Superose 6 10/300 GL column in a buffer containing 20 mM Tris pH 8.0, 300 mM NaCl, and 1 mM TCEP.

### Analytical ultracentrifugation

For centrifugation studies, recombinant HIRA(661–1017), FL CABIN1, and the HIRA/CABIN1 complex were resolved on a Superose 6 10/300 column in 20 mM Tris pH 8.0, 300 mM NaCl, and 1 mM TCEP. Analytical ultracentrifugation experiments were performed with an XL-I analytical ultracentrifuge (Beckman-Coulter) and a TiAn60 rotor with six-channel for sedimentation equilibrium (SE) or two-channel for sedimentation velocity (SV) charcoal-filled epon centerpieces and quartz windows. SE data were collected at 4 °C with detection at 280 nm for 2–3 sample concentrations. SE analyses were carried out using global fits to data acquired at multiple speeds for each concentration with strict mass conservation using the program SEDPHAT, or with the program Heteroanalysis using the ideal fitting model. Error estimates for equilibrium constants derived from SEDPHAT analysis were determined from a 1000-iteration Monte Carlo simulation. See Supplementary Table 1 for a synopsis of loading concentrations, speeds, and model fits.

For SV, absorbance profiles were recorded every 5 min over a period of ~15 h at 141,995×*g*. Data were fit using the *c*(*S*) distribution model of the Lamm equation as implemented in SEDFIT. After optimizing meniscus position and fitting limits, sedimentation coefficients (*S*_T,b_) and frictional ratios (*f/f*_o_) were determined by interactive least-squares fitting of the Lamm equation, with all fit RMSDs less than 0.01. For all analyses, the partial specific volume (*υ*), solvent density (*ρ*), and viscosity (*η*) were derived from chemical composition by SEDNTERP. Hydrodynamic properties calculated from crystal structures were performed using the ZENO extension of the program US-SOMO.

### Crystallographic analysis

Initially HIRA(644–1017) was purified in 300 mM NaCl. However, the purified protein was precipitated during concentration, and it could not be concentrated to higher than 1 mg/ml. HIRA(644–1017) that was purified in 500 mM NaCl (Supplementary Fig. 6a, b) could be concentrated to more than 10 mg/ml and was analyzed by SEC-MALS (Supplementary Fig. 6c; Supplementary Methods), showing that the molecular weight of HIRA(644–1017) was 111,400 Da ± 2.3% in solution. The obtained molecular weight is similar to the theoretical molecular weight of a trimer, 125,815 Da (Supplementary Fig. 6d), and consistent with sedimentation equilibrium analysis of HIRA(661–1017) (Fig. 1e). Crystallization was performed using HIRA(644–1017) (Supplementary Fig. 6c). The initial crystallization screening of HIRA(644–1017) was performed using an automated protein crystallization and monitoring system PXS^46^ with commercially available screening kits (Crystal screen (Hampton research), Crystal screen 2 (Hampton research), PEG/Ion (Hampton research), PEG/Ion 2 (Hampton research), MembFac (Hampton research), Stura FootPrint screen (Molecular Dimensions), Wizard Classic 1 (Rigaku), Wizard Classic 2 (Rigaku), PEGs II suite (QIAGEN), and Protein Complex Suite (QIAGEN). HIRA(644–1017) used in the initial crystallization screening was concentrated to 1.2–19.1 mg/ml. The screening gave crystals from no. 14 of the Stura Footprint (Molecular Dimensions). The crystallization conditions further optimized based on the above conditions by changing the concentration of the precipitant and the value of pH and using additive screen (Hampton research). For the crystallization condition optimization, microseeds of HIRA(644–1017) were also utilized. The best crystal was obtained with reservoir solution of 0.12 M sodium citrate, pH 5.2–5.4, 0.52–0.36 M ammonium sulfate, 0.02 M yttrium(III) chloride; HIRA(644–1017) was crystallized by mixing 2 μl each of 8–10 mg/ml protein and the reservoir solution at 293 K. Se-Met HIRA(644–1017) was crystallized in the same condition of HIRA(644–1017). Microseeds of HIRA(644–1017) were required for the crystallization of Se-Met HIRA(644–1017).

HIRA(644–1017) and Se-Met HIRA(644–1017) crystals were cryo-protected by soaking them in cryo-protectant solution containing 25% (v/v) diethylene glycol, 0.12 M sodium citrate, pH 5.2–5.4, 0.60–0.40 M ammonium sulfate and 0.02 M yttrium(III) chloride for a few seconds. Before X-ray diffraction experiments, crystals were flash-frozen using liquid nitrogen. Se-SAD (single wavelength anomalous diffraction) data of HIRA(644–1017) was collected with an X-ray of wavelength 0.9790 Å at beamline BL41XU (SPring-8, Hyogo, Japan) (Table 1). Native-SAD data of HIRA(644–1017) was collected with an X-ray of wavelength 1.9000 Å at beamline BL-17A at Photon Factory (PF) (KEK, Ibaraki, Japan) (Table 1). Native data of HIRA(644–1017) was collected with an X-ray of wavelength 0.9800 Å at beamline BL-17A (PF) (Table 1). All diffraction data were processed and scaled using the programs XDS and XSCALE^47^, respectively.

Initial phases of the HIRA(644–1017) crystal were obtained by the Se-Met single anomalous diffraction (SAD) method using Phenix.autosol^48^. Initial model building was performed by Phenix.autobuild^48^. While most of the model could be constructed, the model building of the C-terminal region of the HIRA(644–1017) was rather ambiguous. We then obtained the molecular replacement (MR)-native SAD electron density map using Phaser-EP^48^. Importantly, an anomalous difference Fourier map gave positional information of not only Met but also Cys residues, which helped us to construct the molecular model of HIRA(644–1017). Water molecules were added to the coordinates when it satisfied three criteria: Water molecules with *B*-factors were below 80 Å^2^, hydrogen bond formation with other atoms, difference in *B*-factor between water molecule and hydrogen bonding partner atom was below 10 Å^2^. Crystallographic refinement was carried out using Phenix.refine^48^ with diffraction data at 2.45 Å resolution (Table 1).

### Data availability

Coordinates and structure factors for HIRA(644–1017) have been deposited in the Protein Data Bank under the accession code 5YJE. All relevant data is available from the authors upon reasonable request.

## Electronic supplementary material

Supplementary Information

## References

[1] Hammond CM, Stromme CB, Huang H, Patel DJ, Groth A (2017). Histone chaperone networks shaping chromatin function. Nat. Rev. Mol. Cell Biol..

[2] Gurard-Levin ZA, Quivy JP, Almouzni G (2014). Histone chaperones: assisting histone traffic and nucleosome dynamics. Annu. Rev. Biochem..

[3] Tagami H, Ray-Gallet D, Almouzni G, Nakatani Y (2004). Histone H3.1 and H3.3 complexes mediate nucleosome assembly pathways dependent or independent of DNA synthesis. Cell.

[4] Ray-Gallet D (2002). HIRA is critical for a nucleosome assembly pathway independent of DNA synthesis. Mol. Cell.

[5] Ray-Gallet D (2011). Dynamics of histone H3 deposition in vivo reveal a nucleosome gap-filling mechanism for H3.3 to maintain chromatin integrity. Mol. Cell.

[6] Ricketts MD, Marmorstein R (2017). A molecular prospective for HIRA complex assembly and H3.3-specific histone chaperone function. J. Mol. Biol..

[7] Amin AD, Vishnoi N, Prochasson P (2013). A global requirement for the HIR complex in the assembly of chromatin. Biochim. Biophys. Acta.

[8] Tang Y (2006). Structure of a human ASF1a-HIRA complex and insights into specificity of histone chaperone complex assembly. Nat. Struct. Mol. Biol..

[9] Daganzo SM (2003). Structure and function of the conserved core of histone deposition protein Asf1. Curr. Biol..

[10] Shi L, Wen H, Shi X (2016). The histone variant H3.3 in transcriptional regulation and human disease. J. Mol. Biol..

[11] Filipescu D, Muller S, Almouzni G (2014). Histone H3 variants and their chaperones during development and disease: contributing to epigenetic control. Annu. Rev. Cell Dev. Biol..

[12] Buschbeck M, Hake SB (2017). Variants of core histones and their roles in cell fate decisions, development and cancer. Nat. Rev. Mol. Cell Biol..

[13] Adam S, Polo SE, Almouzni G (2013). Transcription recovery after DNA damage requires chromatin priming by the H3.3 histone chaperone HIRA. Cell.

[14] Ricketts MD (2015). Ubinuclein-1 confers histone H3.3-specific-binding by the HIRA histone chaperone complex. Nat. Commun..

[15] Banumathy G (2009). Human UBN1 is an ortholog of yeast Hpc2p and has an essential role in the HIRA/ASF1a chromatin-remodeling pathway in senescent cells. Mol. Cell. Biol..

[16] Sun L (1998). Cabin 1, a negative regulator for calcineurin signaling in T lymphocytes. Immunity.

[17] Jang H, Choi DE, Kim H, Cho EJ, Youn HD (2007). Cabin1 represses MEF2 transcriptional activity by association with a methyltransferase, SUV39H1. J. Biol. Chem..

[18] Jang H, Choi SY, Cho EJ, Youn HD (2009). Cabin1 restrains p53 activity on chromatin. Nat. Struct. Mol. Biol..

[19] Rai TS (2011). Human CABIN1 is a functional member of the human HIRA/UBN1/ASF1a histone H3.3 chaperone complex. Mol. Cell. Biol..

[20] Schneiderman JI, Orsi GA, Hughes KT, Loppin B, Ahmad K (2012). Nucleosome-depleted chromatin gaps recruit assembly factors for the H3.3 histone variant. Proc. Natl Acad. Sci. USA.

[21] Zhang H (2017). RPA interacts with HIRA and regulates H3.3 deposition at gene regulatory elements in mammalian cells. Mol. Cell.

[22] Sarai N (2013). WHSC1 links transcription elongation to HIRA-mediated histone H3.3 deposition. EMBO J..

[23] Soni S, Pchelintsev N, Adams PD, Bieker JJ (2014). Transcription factor EKLF (KLF1) recruitment of the histone chaperone HIRA is essential for beta-globin gene expression. Proc. Natl Acad. Sci. USA.

[24] Zhu Z (2017). PHB associates with the HIRA complex to control an epigenetic-metabolic circuit in human ESCs. Cell Stem Cell.

[25] Lee JS, Zhang Z (2016). O-linked N-acetylglucosamine transferase (OGT) interacts with the histone chaperone HIRA complex and regulates nucleosome assembly and cellular senescence. Proc. Natl Acad. Sci. USA.

[26] Yang JH (2016). Differential regulation of the histone chaperone HIRA during muscle cell differentiation by a phosphorylation switch. Exp. Mol. Med..

[27] De Lucia F (2001). Subnuclear localization and mitotic phosphorylation of HIRA, the human homologue of *Saccharomyces cerevisiae* transcriptional regulators Hir1p/Hir2p. Biochem. J..

[28] Simon AC (2014). A Ctf4 trimer couples the CMG helicase to DNA polymerase alpha in the eukaryotic replisome. Nature.

[29] Guan C, Li J, Sun D, Liu Y, Liang H (2017). The structure and polymerase-recognition mechanism of the crucial adaptor protein AND-1 in the human replisome. J. Biol. Chem..

[30] Tang Y (2012). Identification of an ubinuclein 1 region required for stability and function of the human HIRA/UBN1/CABIN1/ASF1a histone H3.3 chaperone complex. Biochemistry.

[31] Bodor DL, Jansen LE (2013). How two become one: HJURP dimerization drives CENP-A assembly. EMBO J..

[32] Zhu R, Iwabuchi M, Ohsumi K (2017). The WD40 domain of HIRA is essential for RI-nucleosome assembly in Xenopus egg extracts. Cell Struct. Funct..

[33] Villa F (2016). Ctf4 is a hub in the eukaryotic replisome that links multiple CIP-Box proteins to the CMG helicase. Mol. Cell.

[34] Gambus A (2009). A key role for Ctf4 in coupling the MCM2-7 helicase to DNA polymerase alpha within the eukaryotic replisome. EMBO J..

[35] Im JS (2009). Assembly of the Cdc45-Mcm2-7-GINS complex in human cells requires the Ctf4/And-1, RecQL4, and Mcm10 proteins. Proc. Natl Acad. Sci. USA.

[36] Jaramillo-Lambert A (2013). Acidic nucleoplasmic DNA-binding protein (And-1) controls chromosome congression by regulating the assembly of centromere protein A (CENP-A) at centromeres. J. Biol. Chem..

[37] Köhler A, Schmidt-Zachmann MS, Franke WW (1997). AND-1, a natural chimeric DNA-binding protein, combines an HMG-box with regulatory WD-repeats. J. Cell Sci..

[38] Hao J (2015). And-1 coordinates with Claspin for efficient Chk1 activation in response to replication stress. EMBO J..

[39] Weiner MP, Costa GL (1994). Rapid PCR site-directed mutagenesis. PCR Methods Appl..

[40] Sanjana NE, Shalem O, Zhang F (2014). Improved vectors and genome-wide libraries for CRISPR screening. Nat. Methods.

[41] Martini E, Roche DM, Marheineke K, Verreault A, Almouzni G (1998). Recruitment of phosphorylated chromatin assembly factor 1 to chromatin after UV irradiation of human cells. J. Cell Biol..

[42] Clement C, Vassias I, Ray-Gallet D, Almouzni G (2016). Functional characterization of histone chaperones using SNAP-Tag-based imaging to assess de novo histone deposition. Methods Enzymol..

[43] Hall C (2001). HIRA, the human homologue of yeast Hir1p and Hir2p, is a novel cyclin-cdk2 substrate whose expression blocks S-phase progression. Mol. Cell. Biol..

[44] Green CM, Almouzni G (2003). Local action of the chromatin assembly factor CAF-1 at sites of nucleotide excision repair in vivo. EMBO J..

[45] LeMaster DM, Richards FM (1985). 1H-15N heteronuclear NMR studies of *Escherichia coli* thioredoxin in samples isotopically labeled by residue type. Biochemistry.

[46] Hiraki M (2006). Development of an automated large-scale protein-crystallization and monitoring system for high-throughput protein-structure analyses. Acta Crystallogr. D.

[47] Kabsch W (2010). Xds. Acta Crystallogr. D.

[48] Adams PD (2010). PHENIX: a comprehensive Python-based system for macromolecular structure solution. Acta Crystallogr. D.

[49] Winn MD (2011). Overview of the CCP4 suite and current developments. Acta Crystallogr. D.

