# Supplementary material for: Functional activity of the H3.3 histone chaperone complex HIRA requires trimerization of the HIRA subunit
Source: Nat Commun. 2018 Aug 6;9:3103. doi: 10.1038/s41467-018-05581-y (PMC6078998; doi:10.1038/s41467-018-05581-y)
Supplement: Supplementary file 1 — Supplementary Information [file 41467_2018_5581_MOESM1_ESM.pdf]

Supplementary Information

Functional activity of the H3.3 histone chaperone complex HIRA requires trimerization of the HIRA subunit

Ray-Gallet et al.

Supplementary Methods

Protein secondary structure prediction. Predicted secondary structure of HIRA depicted in Supplementary Figure 1a was generated using the PSIPRED secondary structure prediction server [1].

Multiple sequence alignment. Alignment for Supplementary Figure 1b was generated using Clustal Omega [2] and formatted for better visualization using ESPript version 3.0 [3].

SEC-MALS analysis. 20 μl of HIRA(644-1017) (5 mg/ml) was loaded on to WTC-030S5 column (Wyatt) pre-equibrated with 20 mM HEPES-NaOH (pH 6.8), 10% (v/v) glycerol, 500 mM NaCl and 2 mM DTT at 0.5 ml/min. DAWN HELEOS II detector (Wyatt) was used to measure MALS while the RH-101 detecor (Shodex) was used to detect refractive index. Data analysis was performed using ASTRA 6 software (Wyatt).

Supplementary Table1

Sedimentation equilibrium fit results from SEDPHAT

Protein	Loading Concentration (μM)	Rotor speeds (kxg)	Model fit	Mass[a] (kDa)	K_d (μM)	Global Reduced χ^2
HIRA	1.6, 3.2, 6.4	2.9,6.5, 11.6	SS	115,159 ± 621		0.57
			M-Tr	42,951	3.2 ± 0.01	0.61
CABIN1	1.8, 2.7	2.9,6.5, 11.6	SS	245,360 ± 1,586		0.73
HIRA-CABIN1	0.38, 0.64, 0.90	1.3,2.9, 6.5	ABBB[b]	(3:3) 867,780	K_d1 = 0.002 K_d2 = 0.002 K_d3 = 9.6	1.12
			ABB	(3:2) 621,471	K_d1 = <0.001 K_d2 = <0.001	1.31

M = monomer, Tr = trimer, SS = single species

a. Errors were determined by 1000 iterations of Monte-Carlo analysis
b. Where A is a trimer of HIRA and B is a monomer of CABIN1

Supplementary Table 2

Sedimentation equilibrium fitting for HIRA trimer in SEDPHAT

Fixed LOG(Ka)	X^2	Kd (μM)	Residuals
9	2.81		poor
9.5	1.5	17.7	poor
10	0.872	10	so-so
11	0.612	3	ok
12	0.713	1	ok
13	0.823	0.3	ok
14	0.881	0.1	so-so
15	0.915	0.031	poor

Float LOG(Ka)	X^2	Kd (μM)	Residuals
10.904	0.61	3.6	best

Supplementary References

1. Buchan, D.W., Minneci, F., Nugent, T.C., Bryson, K. & Jones, D.T. Scalable web services for the PSIPRED Protein Analysis Workbench. *Nucleic Acids Res.* **41**, W349-W357 (2013).

2. Sievers, F. & Higgins, D.G. Clustal Omega, accurate alignment of very large numbers of sequences. *Methods Mol. Biol.* **1079**, 105-116 (2014).

3. Gouet, P., Robert, X. & Courcelle, E. ESPript/ENDscript: Extracting and rendering sequence and 3D information from atomic structures of proteins. *Nucleic Acids Res.* **31**, 3320-3323 (2003).

a Predicted secondary structure of the human HIRA protein

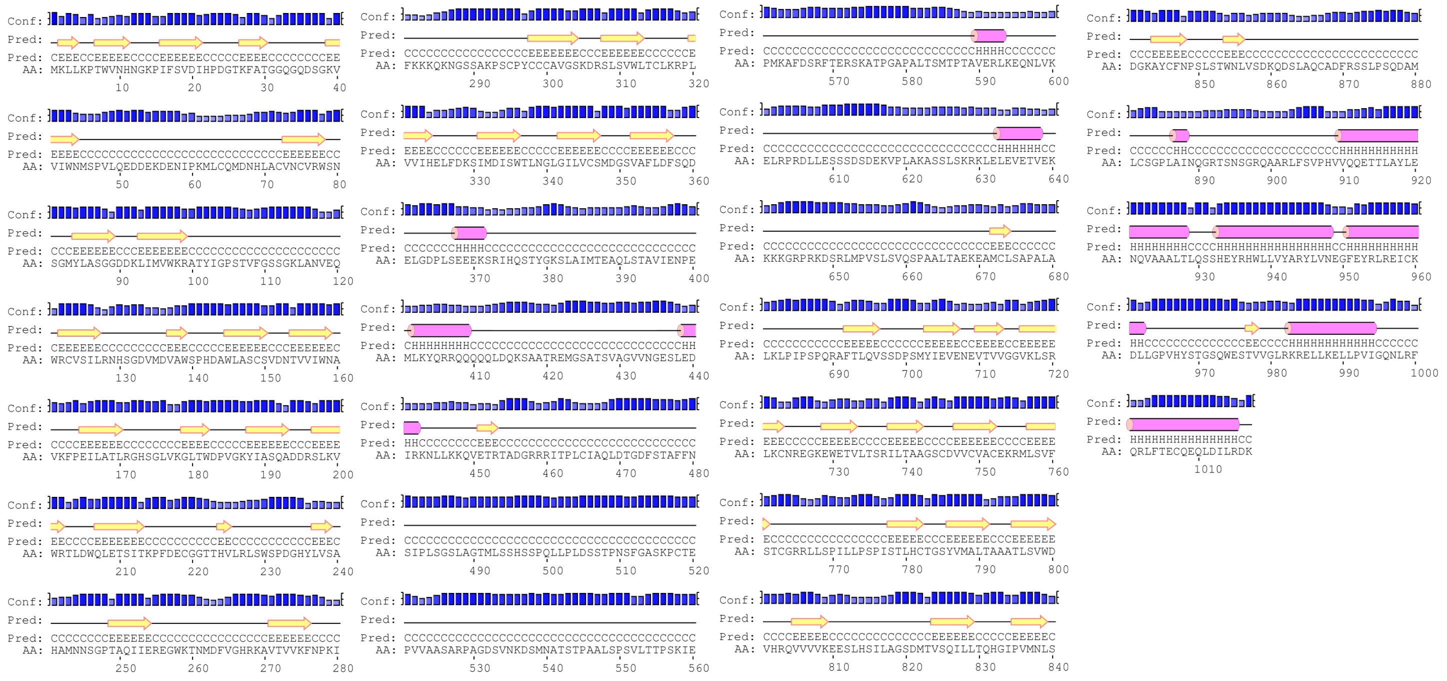

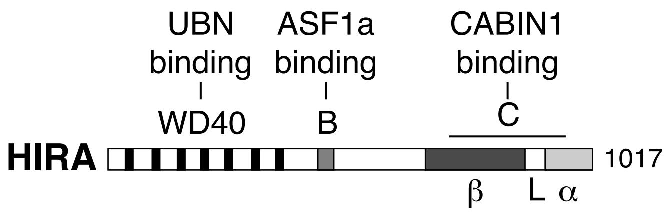

b Alignment of HIRA amino acid sequences

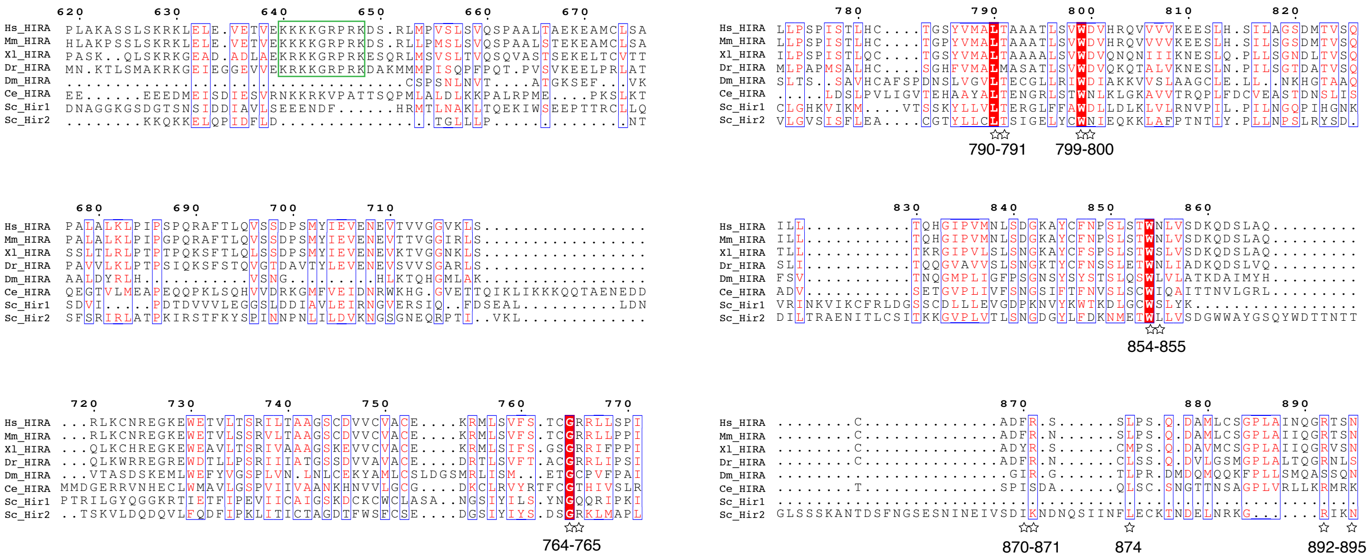

Supplementary Figure 1. HIRA secondary structure and sequence conservation. **a** Predicted secondary structure for the human HIRA protein generated using the PSIPRED server (http://bioinf.cs.ucl.ac.uk/psipred/). Three different structures matching the C domain are identified: a β-strand (aa 661-872), a loop (aa 873-904) and a α-helical (aa 905-1017) domains. **b** Multiple sequence alignment depicting conservation among *Homo sapiens* (Hs) HIRA, *Mus musculus* (Mm) HIRA, *Xenopus laevis* (Xl) HIRA, *Danio rerio* (Dr) HIRA, *Drosophila melanogaster* (Dm) HIRA, *Caenorhabditis elegans* (Ce) HIRA, *Saccharomyces cerevisiae* (Sc) Hir1, and *Saccharomyces cerevisiae* (Sc) Hir2. Alignment represents human HIRA residues 619-895 and was performed with Clustal Omega and formatted for visualization with ESPript version 3.0. Highly conserved amino acids are shown in red boxes. Star indicates amino acid subjected to mutagenesis in our study. The conserved AT-hook motif (aa 640-648) is boxed in green.

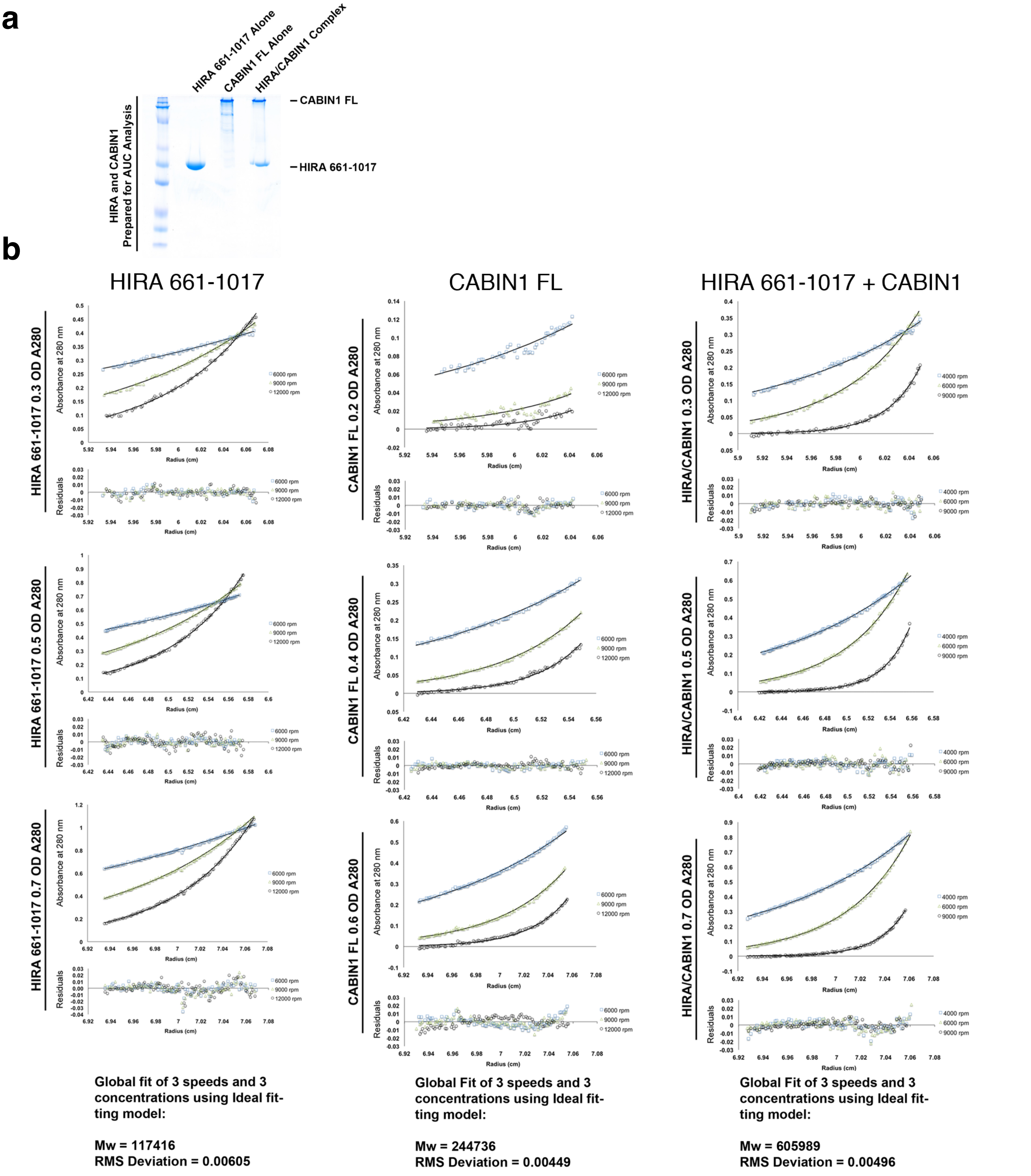

Supplementary Figure 2. HIRA sedimentation properties. a Representative gel of analytical ultracentrifuge input samples for HIRA(661-1017), CABIN1 FL, and HIRA-CABIN1 complex from size-exclusion chromatography. **b** (Left) Sedimentation equilibrium performed with HIRA(661-1017) alone, three protein concentrations (0.3, 0.5, and 0.7 OD A280) were analyzed at three speeds (2898, 6520, and 11591 xg). (Middle) Sedimentation equilibrium performed with CABIN1 alone, three protein concentrations (0.2, 0.4, and 0.6 OD A280) were analyzed at three speeds (2898, 6520, and 11591 xg). (Right) Sedimentation equilibrium performed with the HIRA-CABIN1 complex, three different protein concentrations (0.3, 0.5, and 0.7 OD A280) were analyzed at three different speeds (1288, 2898, and 6520 xg). These data were fit using the ideal fitting model with the program HeteroAnalysis.

c

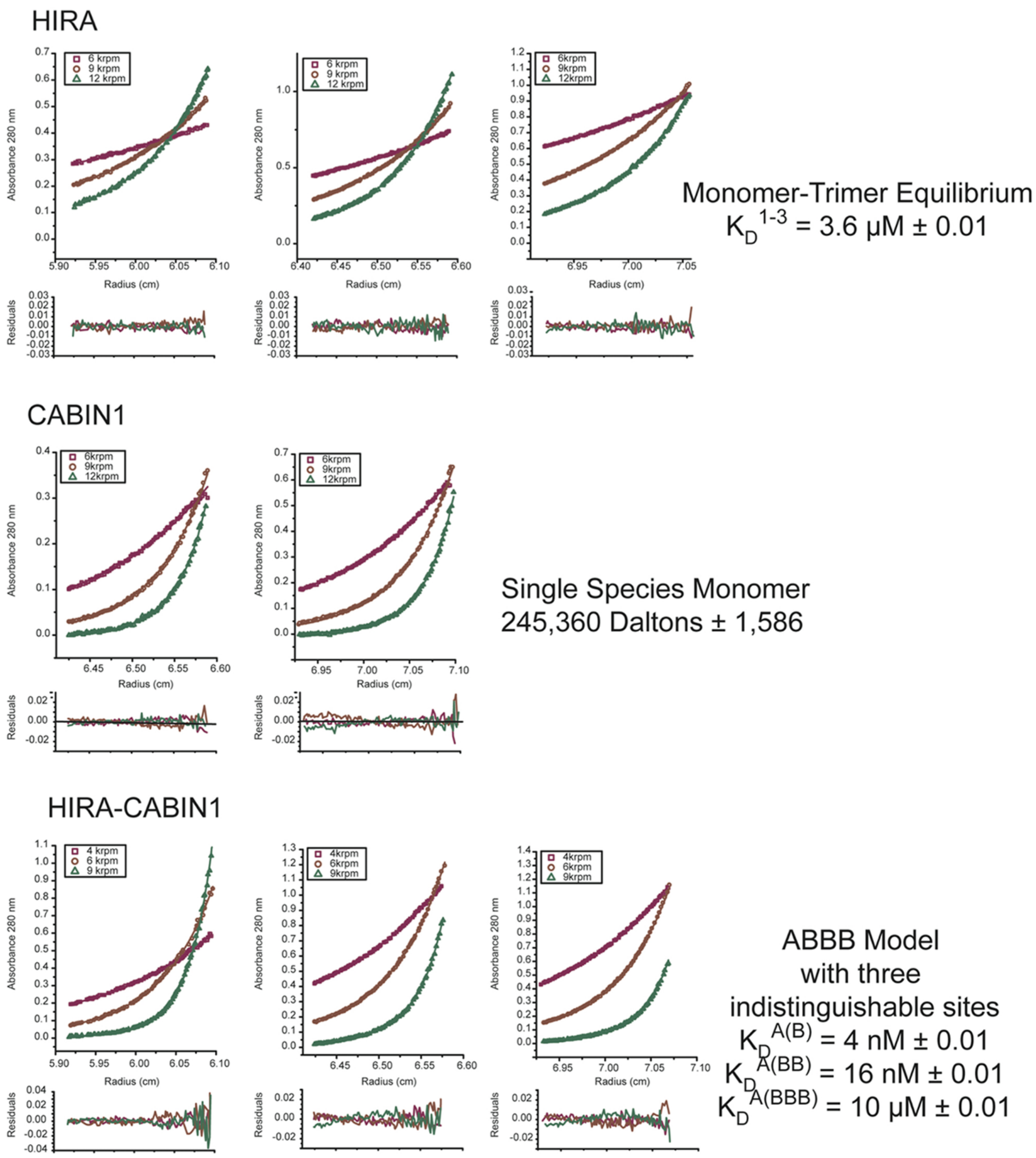

d

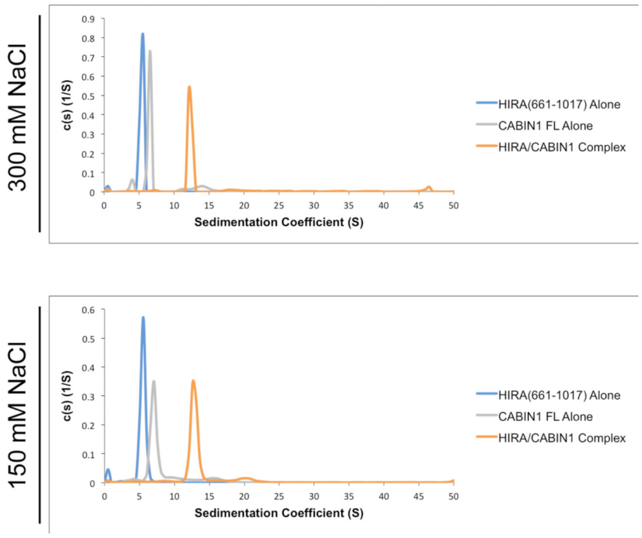

Supplementary Figure 2. HIRA sedimentation properties. **c** Sedimentation equilibrium analysis of HIRA(661-1017), CABIN1 (FL) and the HIRA-CABIN1 complex under the same experimental conditions but fit using the program SEDPHAT. **d** Sedimentation velocity analysis for HIRA(661-1017), CABIN1 FL, and the HIRA-CABIN1 complex conducted at a centrifugation speed of 141,995 xg . Proteins were analyzed in buffer containing either 300 mM (top) or 150 mM NaCl (bottom). Data were analyzed using SEDFIT to generate a continuous c(s) distribution to monitor the presence of discrete populations in solution.

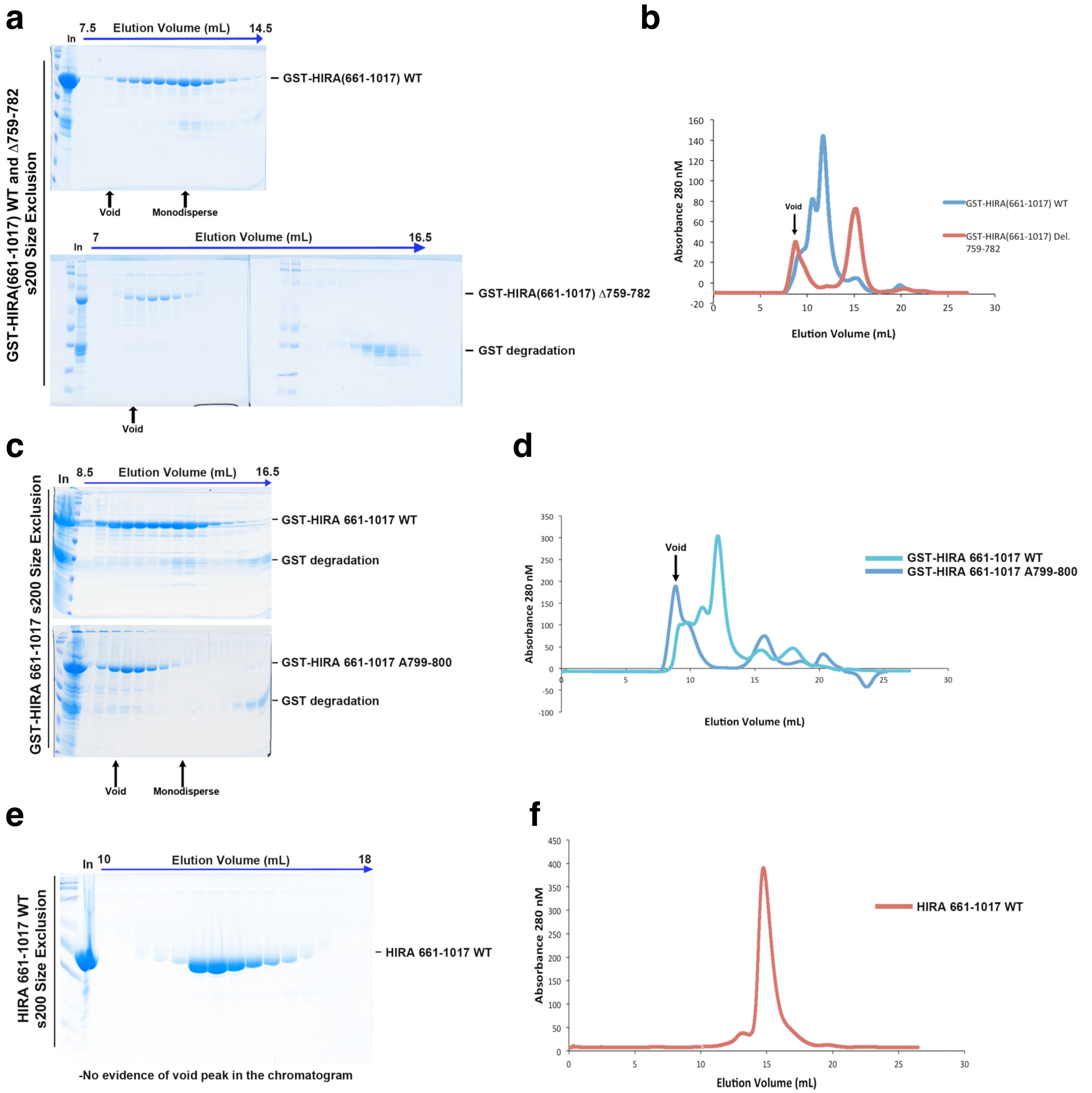

Supplementary Figure 3. Size exclusion analysis of HIRA(661-1017) constructs. a Superdex 200 fractionation of GST-HIRA(661-1017) WT and GST-HIRA(661-1017) Δ759-782. **b** Superdex 200 size-exclusion chromatograms of GST-HIRA(661-1017) WT and GST-HIRA(661-1017) Δ759-782. **c** Superdex 200 fractionation of GST-HIRA(661-1017) WT and GST-HIRA(661-1017) W799A-D800A. **d** Superdex 200 size-exclusion chromatograms of GST-HIRA(661-1017) WT and GST-HIRA(661-1017) W799A-D800A. **e** Superdex 200 fractionation of HIRA(661-1017) WT. **f** Superdex 200 size-exclusion chromatograms of HIRA(661-1017) WT.

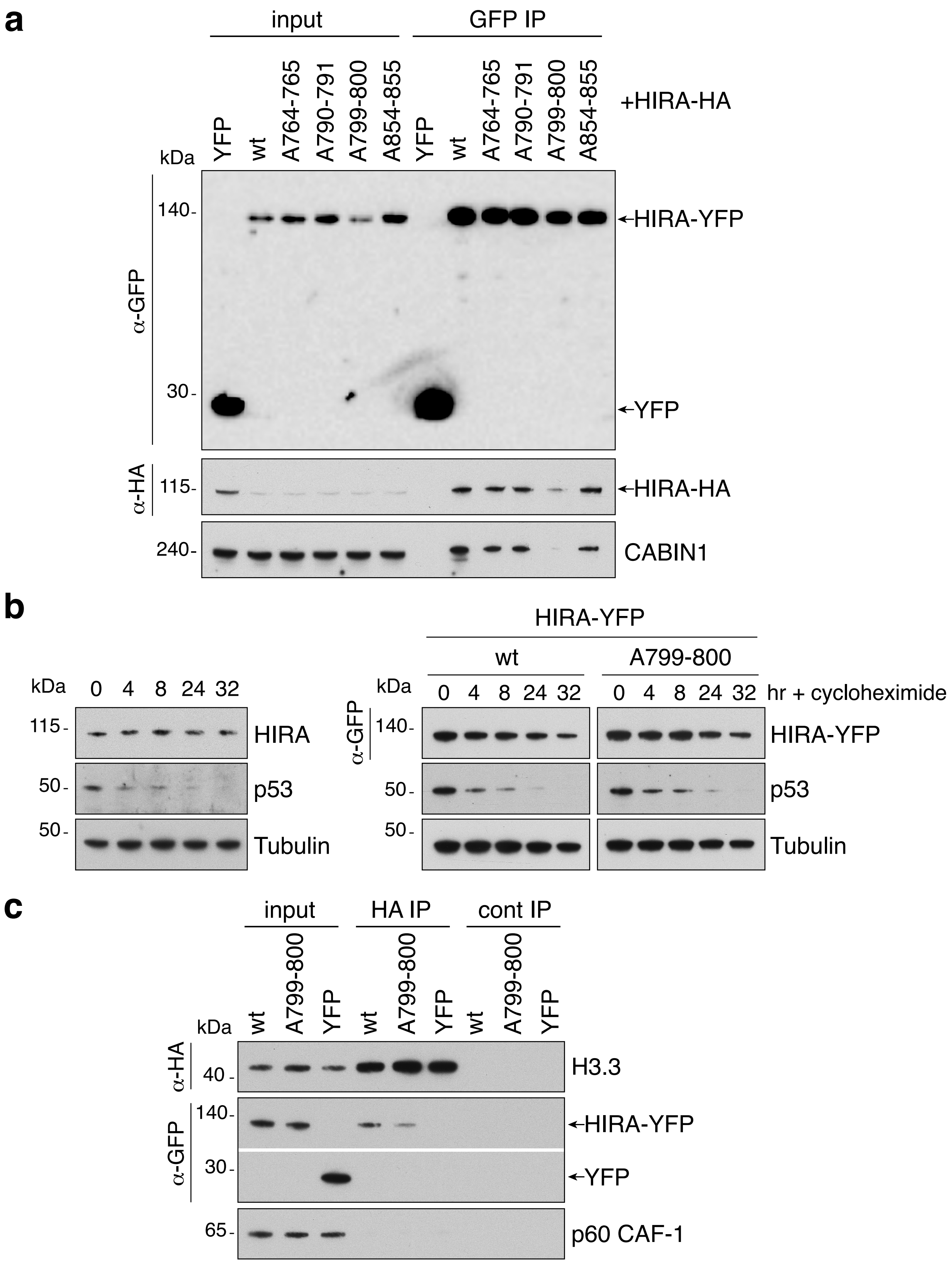

Supplementary Figure 4. a HIRA(W799A-D800A) interacts less efficiently with CABIN1 than the HIRA wt protein. We used nuclear extracts from U2OS cells expressing both YFP-tagged and HIRA-HA proteins for anti-GFP immunoprecipitation and showed the corresponding Western blots. Input corresponds to 10% of nuclear extract used for each experiment. **b Comparable protein stability for HIRA-YFP wt and mutant (W799A-D800A)**. Non-transfected (Left) and transfected (Right) U2OS cells were treated with cycloheximide (50 μg/ml) during the indicated times before preparing whole-cell extracts and analysis by Western blotting. **c Both HIRA-YFP wt and mutant (W799A-D800A) proteins interact with H3.3.** We performed anti-HA or control immunoprecipitations using nuclear extracts from transfected HeLa H3.3-SNAP-HA cells expressing either YFP, HIRA-YFP wt or mutant (W799A-D800A). The co-immunoprecipitation of YFP proteins with H3.3-SNAP-HA was analyzed by Western blotting. Input corresponds to 10% of nuclear extract used for each experiment.

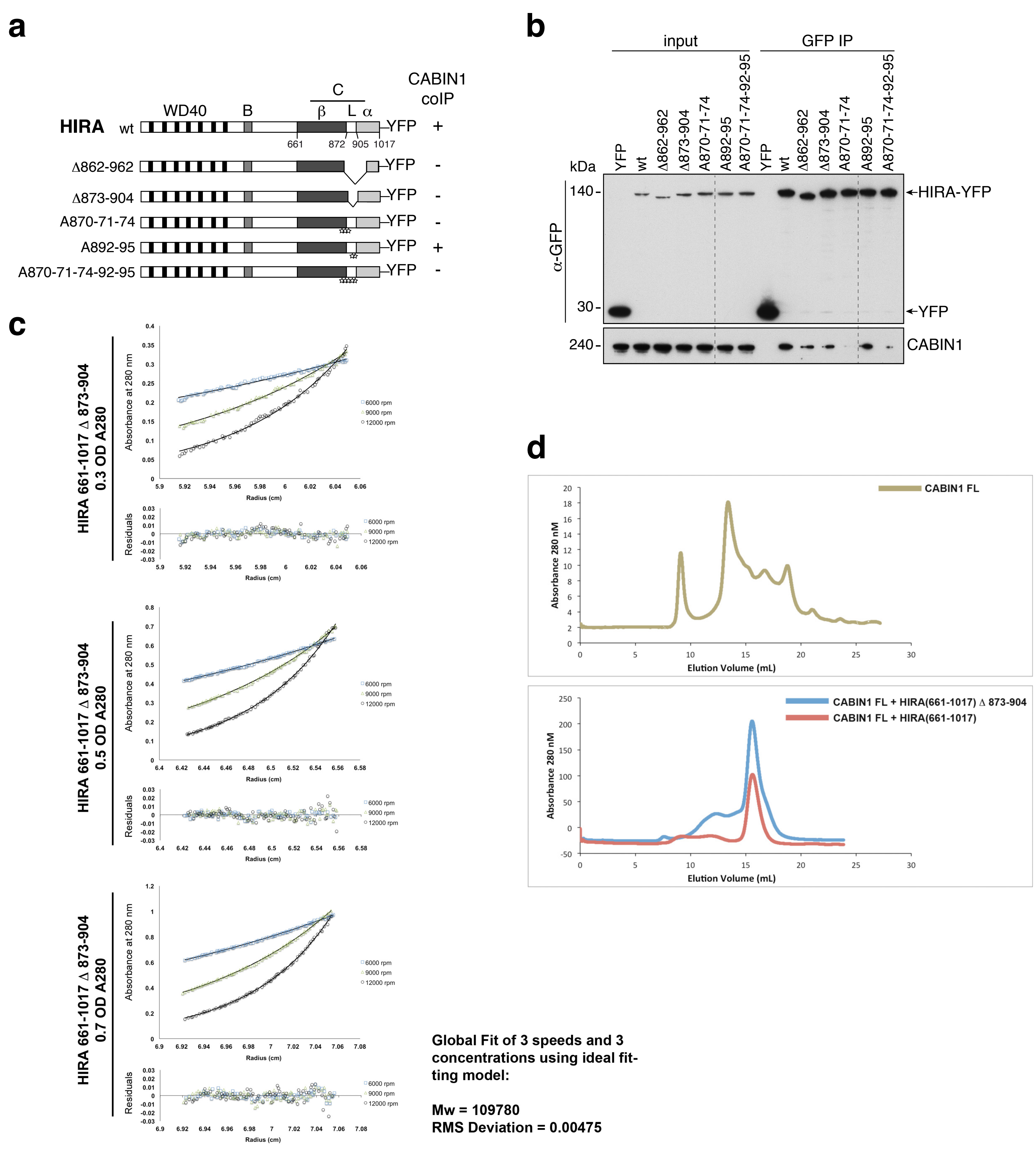

Supplementary Figure 5. **HIRA interaction with CABIN1: co-immunoprecipitation, sedimentation equilibrium and size-exclusion analysis. a** YFP constructs of human HIRA and mutants. Star indicates single amino acid substitution with alanine. The co-immunoprecipitation efficiency of endogenous CABIN1 (CABIN1 coIP) is indicated for each construct, « + » means that the efficiency of coIP is similar to the one obtained with the wt HIRA construct and « - » means that the efficiency of the coIP is decreased. **b** Western blot analysis of anti-GFP-immunoprecipitates from U2OS nuclear extracts expressing YFP-tagged proteins. Input corresponds to 10% of nuclear extract used for each experiment. **c** Sedimentation equilibrium performed with HIRA(661-1017) Δ873-904 alone, three protein concentrations (0.3, 0.5, and 0.7 OD A280) were analyzed at three centrifugation speeds (6000, 9000, and 12000 RPM). **d** Superose 6 size-exclusion chromatograms of CABIN1 FL, CABIN1 FL + HIRA(661-1017) and CABIN1 FL + HIRA (661-1017) Δ873-904 related to figure 3d.

e

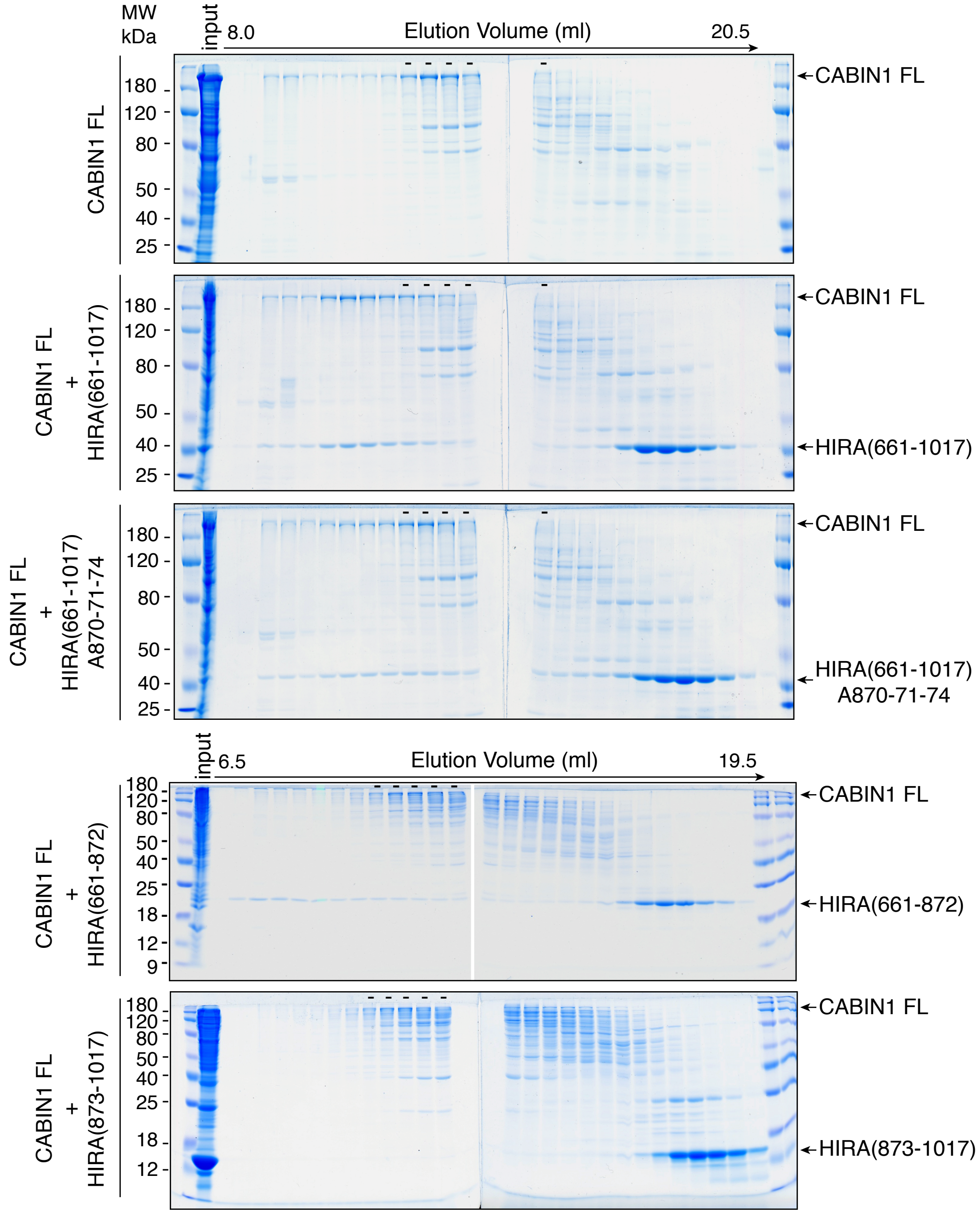

f

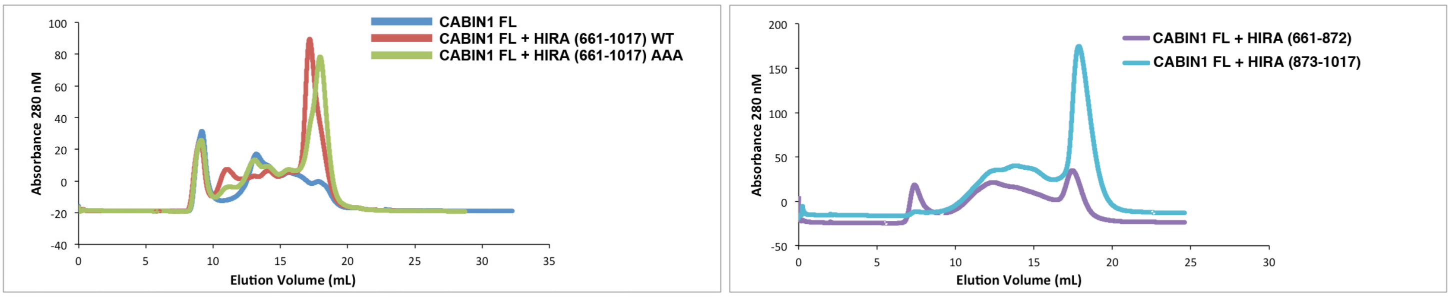

Supplementary Figure 5. HIRA interaction with CABIN1: co-immunoprecipitation, sedimentation equilibrium and size-exclusion analysis. e Superose 6 fractionation of recombinant protein CABIN1 FL in combination with different HIRA constructs: aa 661-1017 (β-strand+loop+α-helical), aa 661-1017 mutant (FRL870-71-74AAA), aa 661-872 (β-strand+loop) and aa 873-1017 (loop+α-helical). The dashes indicate the fractions in which free CABIN1 FL elutes. Input corresponds to protein at the concentration that it was loaded onto the Superose 6 column.
f Superose 6 size-exclusion chromatograms of CABIN1 FL, CABIN1 FL + HIRA(661-1017) and CABIN1 FL + HIRA(661-1017) (FRL870-71-74AAA) (Left) and of CABIN1 FL + HIRA(661-872) and CABIN1 FL + HIRA(873-1017) (Right).

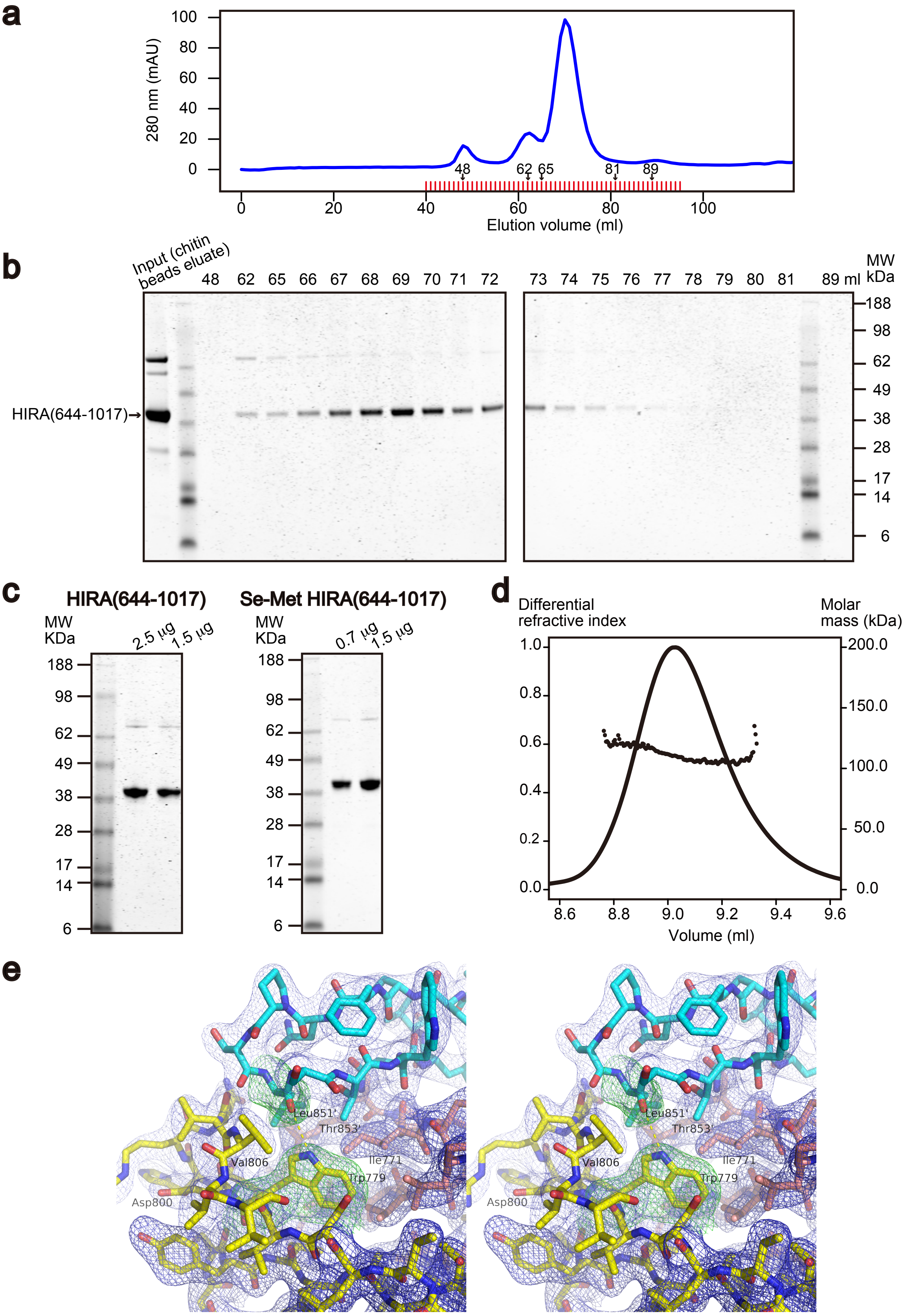

Supplementary Figure 6. **Size-exclusion analysis and homotrimer formation of HIRA(644-1017). a** HiLoad 16/600 Superdex 200 pg size-exclusion chromatograms for HIRA(644-1017). **b** HiLoad 16/600 Superdex 200 pg fractionations of HIRA(644-1017). **c** Representative SDS-PAGE gel of HIRA(644-1017) and Se-Met HIRA(644-1017) used for SEC-MALS or crystallization. **d** SEC-MALS analysis for HIRA(644-1017). Line indicates measured differential refractive index (left axis) and dots indicated molar masses (right axis). **e** The electron density maps showing the stereo image of HIRA(644-1017). 2mFo-DFc electron density map (blue) of the interface between subunits A (yellow and pink) and B (cyan) of HIRA(644-1017) at 2.45 Å resolution. The density was contoured at 1σ. The mFo-DFc simulated annealing (SA)-omit map for Trp799 in subunit A and Leu851 in subunit B is shown in green. The density was contoured at 3σ, Yellow dashed line indicates the interaction between Trp799 and Leu851.

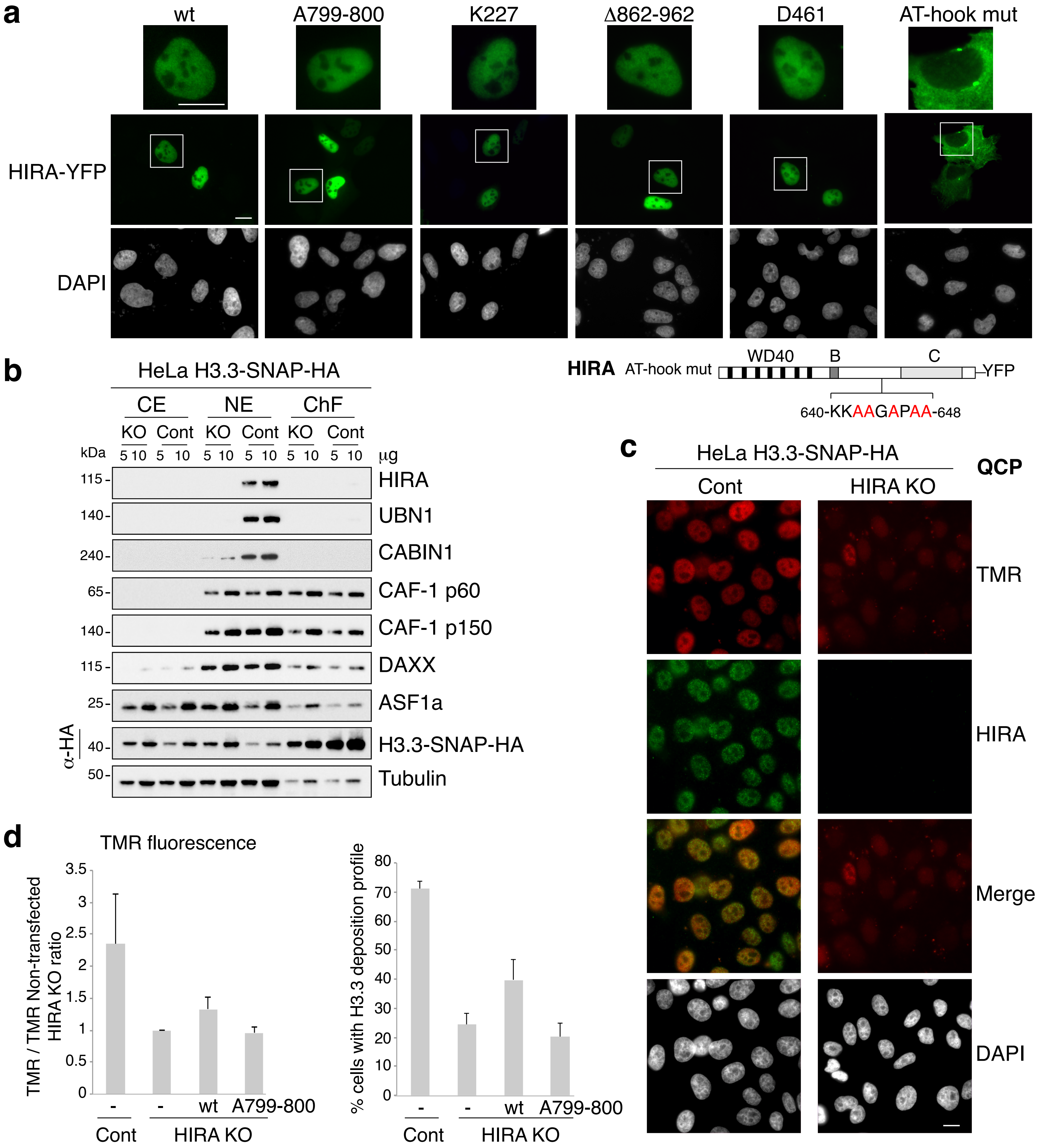

Supplementary Figure 7. Expression and activity of HIRA wt and mutants in cells. a Expression of HIRA-YFP constructs in U2OS cells. Enlarged images of one selected cell are presented. Scale bar, 10 μm. At the bottom right, a scheme of the AT-hook mutant is shown. Amino acids substituted with alanines are indicated in red. **b** Western blot analysis of cytosolic extracts (CE), nuclear extracts (NE) and chromatin fractions (ChF) from control and HIRA KO HeLa H3.3-SNAP-HA cells. **c** Visualization of new H3.3 deposition (TMR) by Quench-Chase-Pulse (QCP) experiment and endogenous HIRA by immunofluorescence in control and HIRA KO HeLa H3.3-SNAP-HA cells. **d** Graphs of the QCP experiments shown in figure 5b including the results for the control HeLa H3.3-SNAP-HA cell line.

a

b

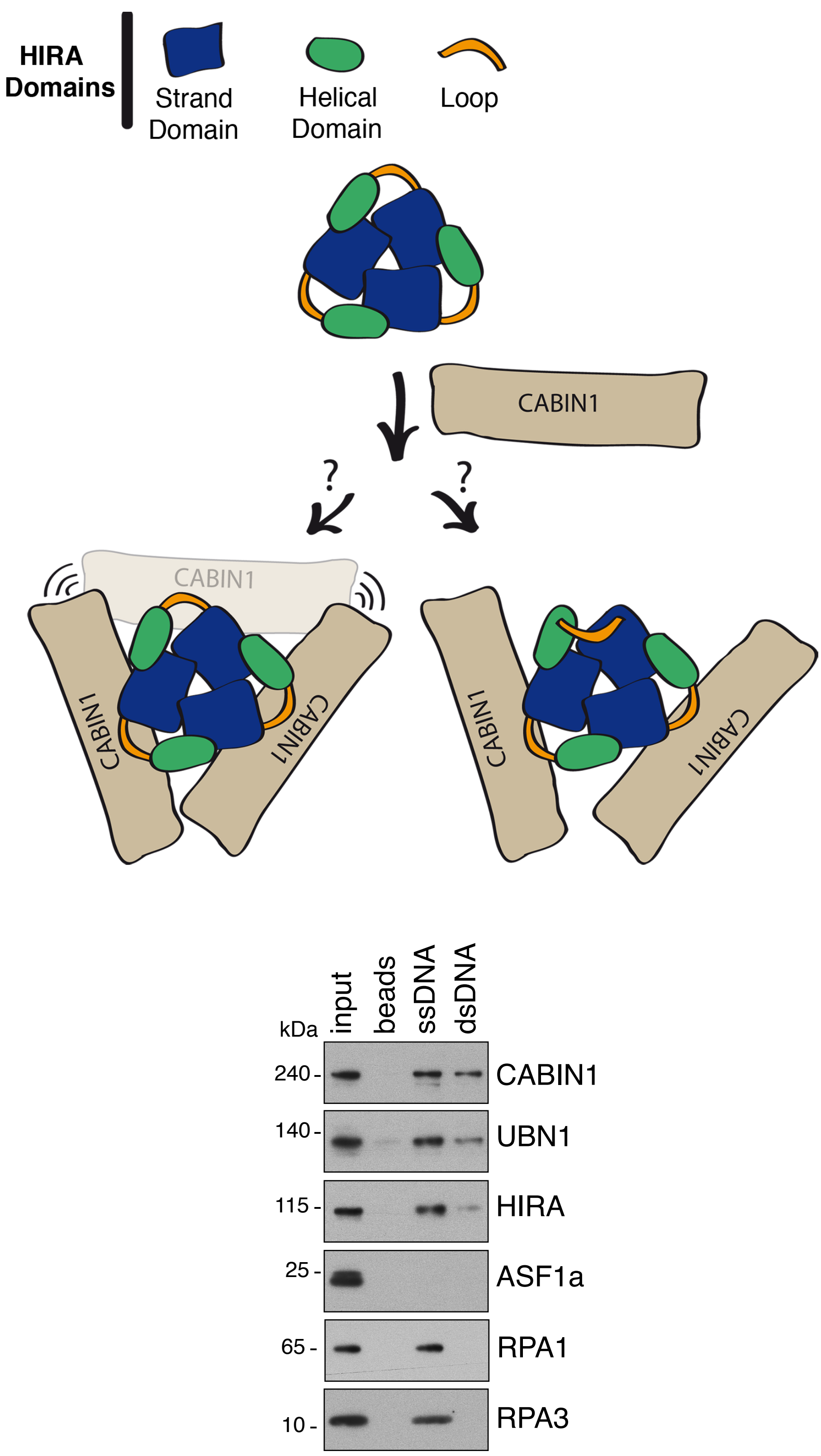

Supplementary Figure 8. a Two schematic models of the 3(HIRA):2(CABIN1) complex formation. The first model is based on the fact that the third molecule of CABIN1 is unable to bind HIRA due to steric occlusion (Left). The second one proposes that the third molecule of CABIN1 is unable to bind due to one HIRA loop being burried, or another structural rearrangement making the third HIRA subunit inaccessible for CABIN1 binding (Right). **b The HIRA complex binds both ds and ssDNA.** dsDNA fixed on magnetic beads was treated with NaOH 0.2 M to get ssDNA fixed on magnetic beads. For the DNA binding assay, mock, dsDNA or ssDNA beads were incubated with 16 μg of nuclear extracts from U2OS cells and bound proteins were analyzed by Western blotting. RPA1 and RPA3 blots served as ssDNA binding protein controls.

for Fig.1b

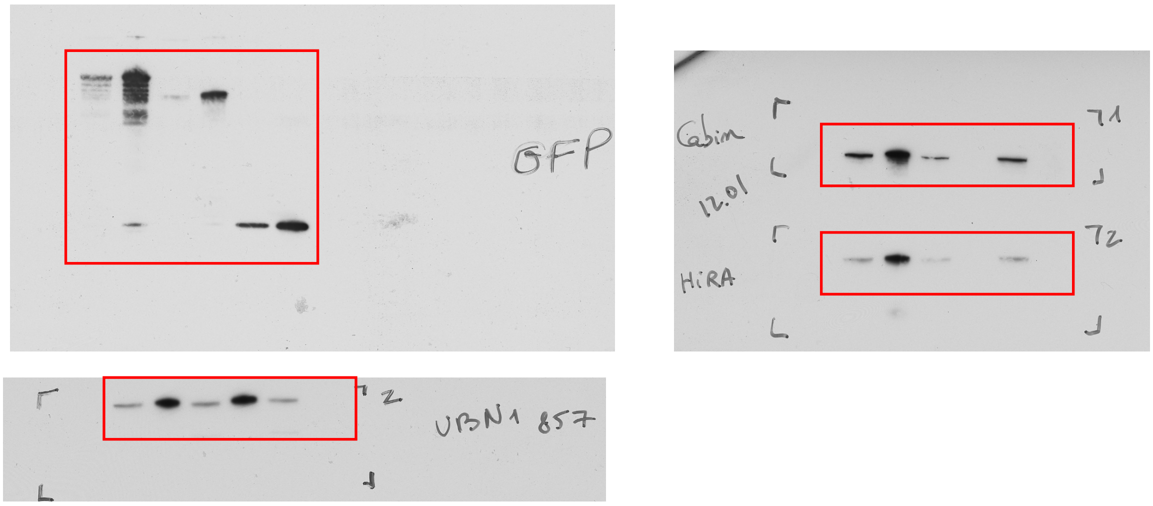

for Fig.1c

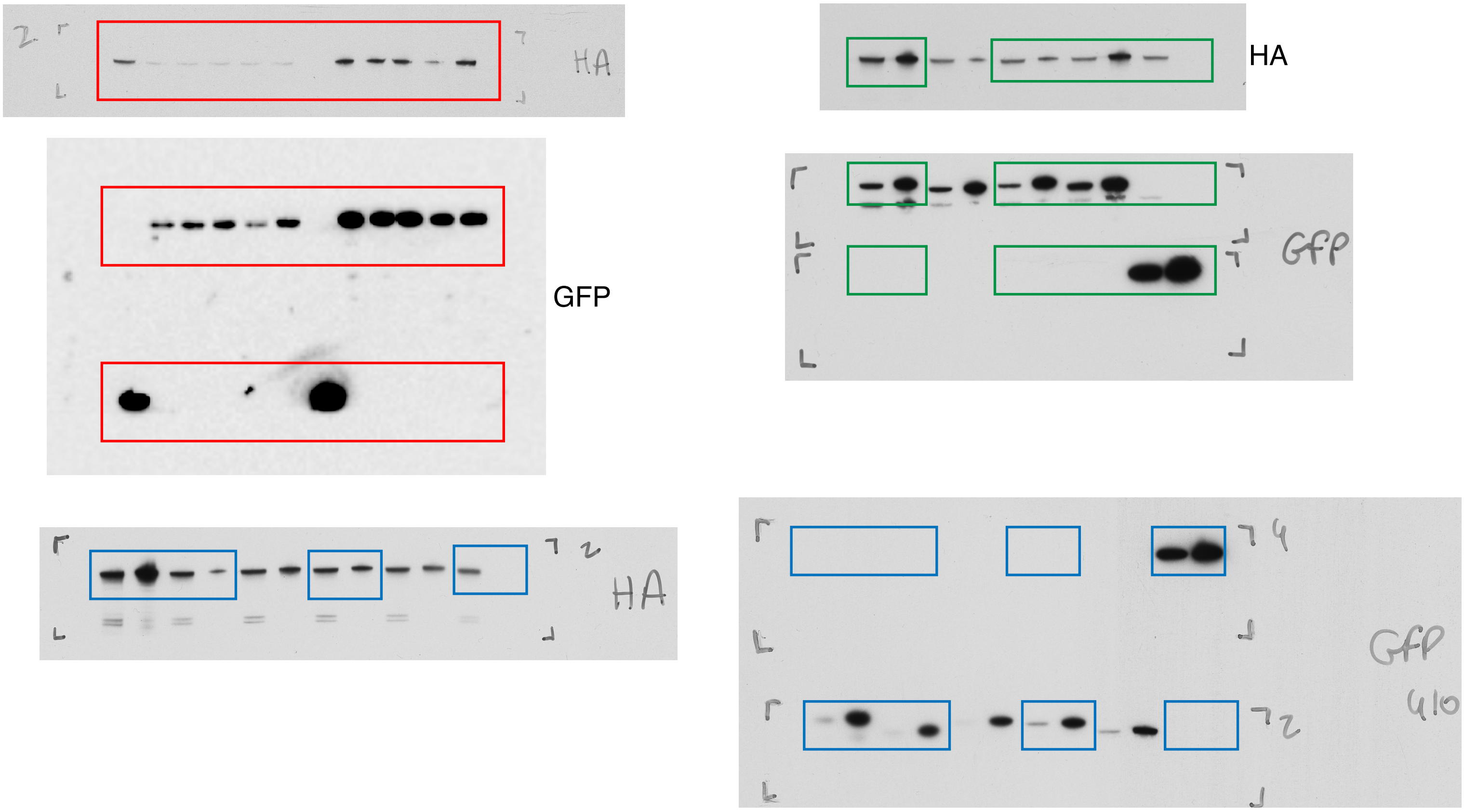

for Fig.1d

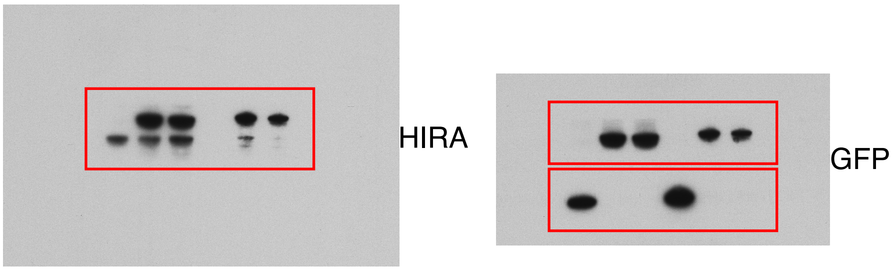

Supplementary Figure 9. Uncropped Western blots for Fig.1

for Fig. 2b

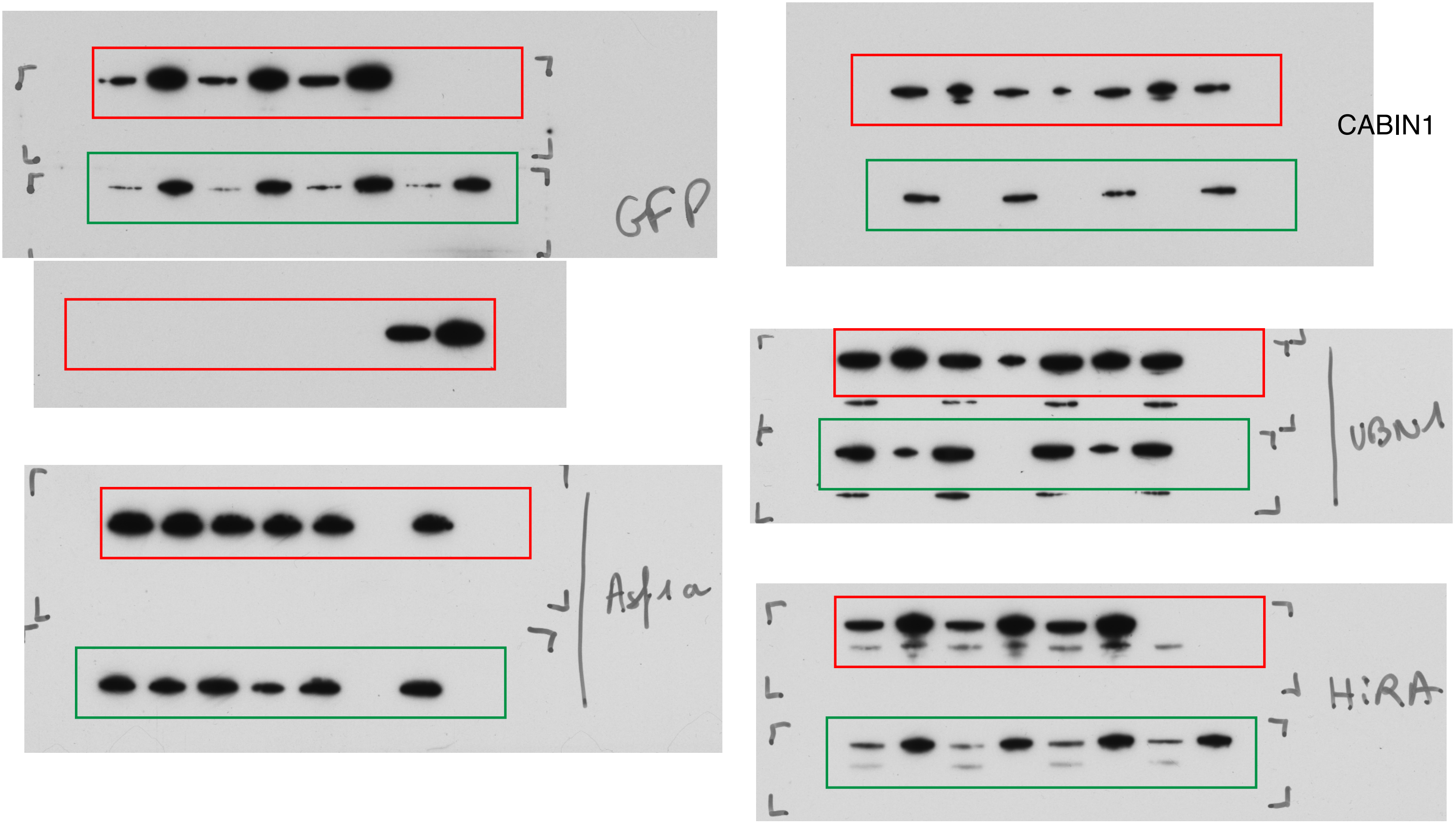

for Fig. 2c

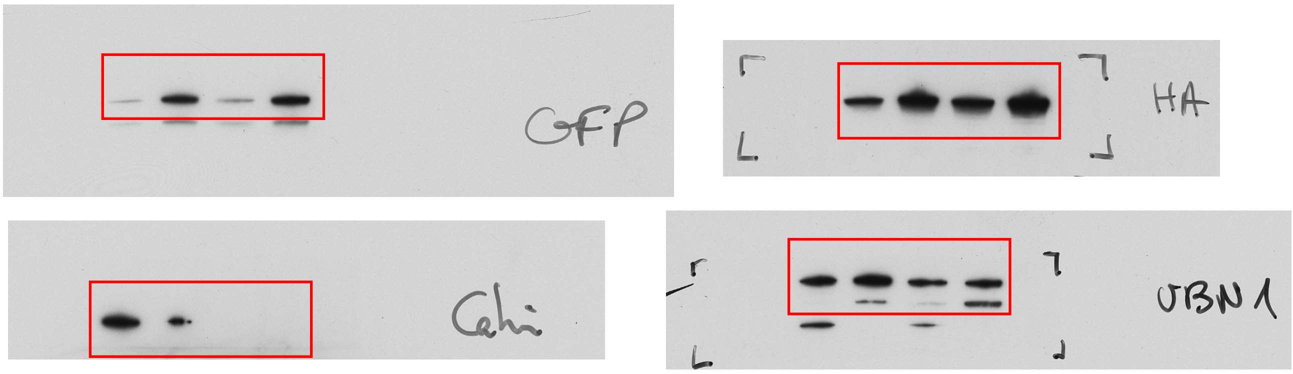

Supplementary Figure 10. Uncropped Western blots for Fig. 2

for Fig. 3b

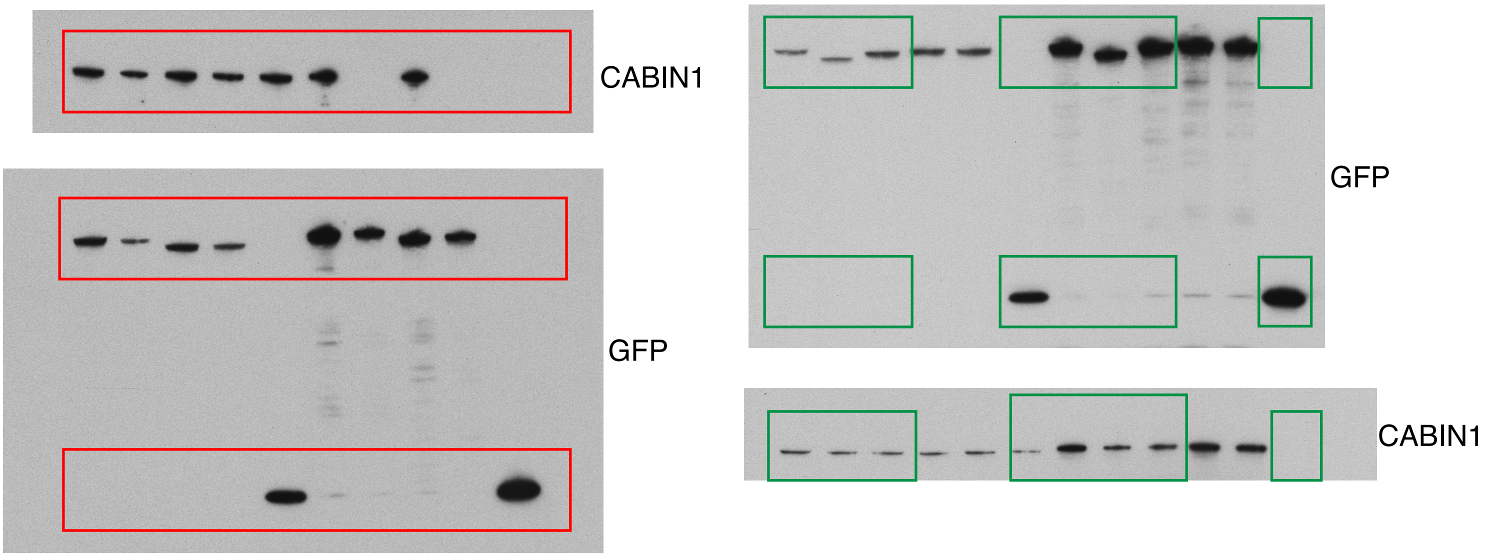

for Fig. 3c

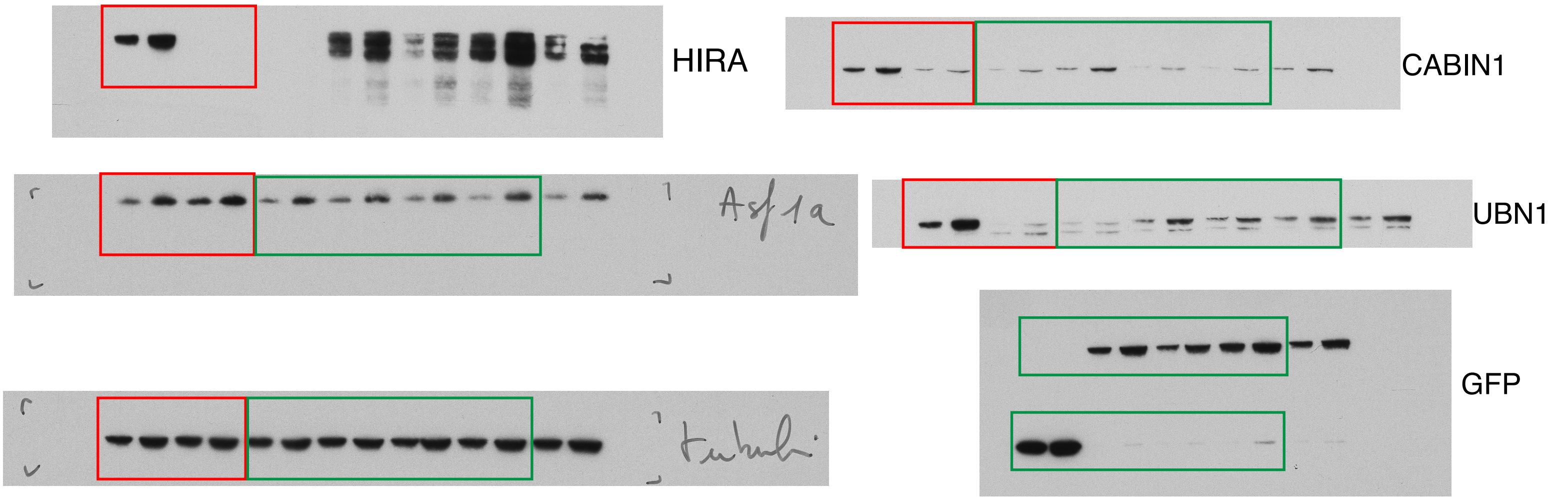

for Fig. 6b

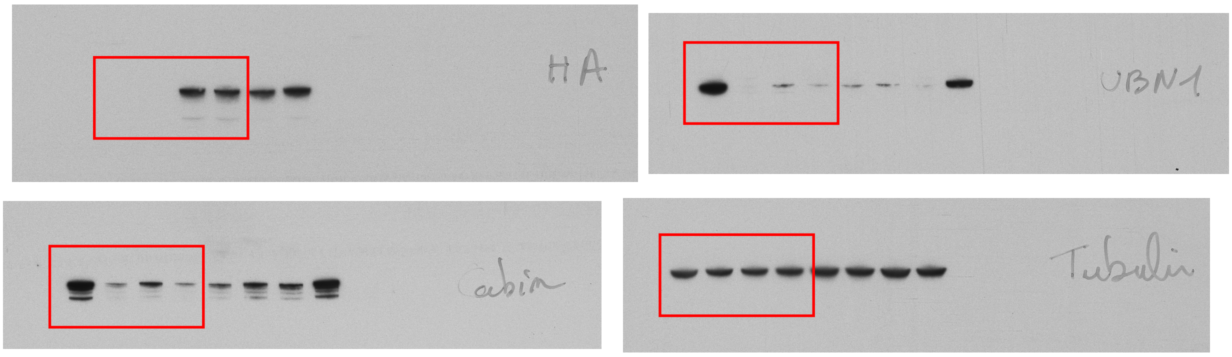

Supplementary Figure 11. Uncropped Western blots for Fig. 3 and Fig. 6
